# Establishment and Parameter Calibration of a Discrete Element Model for Shanghai Bok Choy Plug Seedling

**DOI:** 10.3390/plants15121882

**Published:** 2026-06-17

**Authors:** Jiawei Shi, Jianping Hu, Wei Liu, Ji Chen, Che Wang, Mengjiao Yao

**Affiliations:** 1 Jiangsu Provincial Key Laboratory of Hi-Tech Research for Intelligent Agricultural Equipment, Jiangsu University, Zhenjiang 212013, China; 2112116016@stmail.ujs.edu.cn (J.S.); mario_liu@ujs.edu.cn (W.L.); 2222316032@stmail.ujs.edu.cn (C.W.); 2112216018@stmail.ujs.edu.cn (M.Y.); 2 Shanghai Agricultural Machinery Research Institute, Shanghai 201106, China; shnjscj@163.com

**Keywords:** Shanghai bok choy plug seedling, discrete element method, material properties, parameter calibration, automatic transplanting

## Abstract

To address the significant differences in the structure and mechanical properties of various components of the Shanghai bok choy plug seedling, and the lack of an accurate and reliable discrete element model of the whole plant and key bonding parameters in the simulation of the automatic transplanting process, a 128-cell Shanghai bok choy plug seedling was selected as the research object. Morphological, physical, mechanical, and contact property tests were systematically conducted to obtain the basic parameters of the seedling pot, leaf, petiole, and stem. A whole-plant discrete element model of Shanghai bok choy plug seedling, consisting of the seedling pot, leaf, petiole, and stem, was established using a combined method of component-wise modeling and overall reconstruction. The Hertz–Mindlin (no slip) and Bonding V2 contact models were jointly adopted to characterize interparticle contact, continuous structural behavior, and failure characteristics. Taking the ultimate compressive failure load of the seedling pot, leaf compression density, ultimate bending failure load of the petiole, and ultimate bending failure load of the stem as response indices, significant parameters were screened using the Plackett–Burman test, the optimization ranges were determined through the steepest ascent test, and the key bonding parameters were optimized and calibrated using the Box–Behnken response surface test. The results showed that the relative errors between the simulated and experimental values of the ultimate compressive failure load of the seedling pot, leaf compression density, ultimate bending failure load of the petiole, and ultimate bending failure load of the stem after optimization were 1.19%, 1.13%, 0.99%, and 0.72%, respectively, indicating that the established model can accurately characterize the mechanical response of the constituent parts of Shanghai bok choy plug seedling. The results provide a basis for discrete element simulation of the interaction between Shanghai bok choy plug seedling and key components of automatic transplanting equipment, as well as for the design optimization of automatic transplanting equipment.

## 1. Introduction

Shanghai bok choy is a high-quality leafy vegetable widely cultivated in southern China. It is characterized by a short growth cycle, a high multiple cropping index, and strong market demand, and plays an important role in ensuring the year-round stable supply of vegetables [1,2,3,4,5]. With the continuous expansion of large-scale and intensive Shanghai bok choy cultivation, higher requirements have been imposed on the efficient, low-damage, and stable mechanical transplanting of plug seedlings. Greater attention has also been paid to the rationality of transplanting equipment, structural design and the scientific matching of operating parameters [6,7,8,9]. Traditional mechanical design methods mainly rely on empirical design and repeated testing, resulting in a relatively long research and development cycle and difficulty in meeting the demands for rapid optimization and technological upgrading of vegetable transplanting equipment [10,11,12,13,14]. Owing to its advantages in simulating contact deformation of discontinuous media, the discrete element method has been increasingly applied in agricultural engineering [15,16,17]. The discrete element method is of great significance for the design of key components and optimization of operating parameters of Shanghai bok choy automatic transplanting equipment, while the construction of an accurate discrete element model of Shanghai bok choy plug seedling and the calibration of key parameters are fundamental to ensuring the reliability of simulation results. Establishing a discrete element model of Shanghai bok choy plug seedling and revealing its interaction with key contact components, such as the seedling picking mechanism, seedling separating mechanism, and planting mechanism, through simulation analysis can provide a theoretical basis for the structural design and parameter optimization of automatic transplanting equipment.

As an important method for analyzing the operation process of agricultural equipment, the discrete element method can establish reliable discrete element models of materials through parameter calibration, thereby effectively simulating the dynamic contact and interaction between agricultural equipment and working materials [18,19,20,21,22]. In recent years, researchers worldwide have developed various agricultural material models based on discrete element technology, laying a foundation for equipment–material interaction simulation. Lenaerts et al. [23] established a flexible discrete element model of wheat straw using a bonded discrete element model, in which adjacent particles were connected by adjusting the mechanical properties of the bonds. The feasibility of the model was verified by calibrating parameters such as the elastic modulus and simulating the bending behavior of straw through a three-point bending test. Kwork et al. [24] systematically analyzed the applicability of different combinations of discrete element contact models in soil tillage simulation and verified them using the angle of repose test. Several model combinations capable of effectively characterizing normal, tangential, and cohesive interactions in soil were selected, providing a reference for contact model selection in agricultural machinery–soil interaction simulation. Kim et al. [25] established a soil model considering the properties of different soil layers based on the discrete element method, and completed parameter calibration using virtual rotary shear tests and cone penetration tests. Field test validation showed that the model could accurately predict tillage draft resistance at different tillage depths, providing an effective method for design load prediction of agricultural machinery. Zhang et al. [26] established a heterogeneous cultivated-layer model with stratified differences in aggregate structure based on the Hertz–Mindlin with JKR model, and identified the key parameters affecting the rheological properties of heavy clay using the angle of repose and triaxial compression tests. Shen et al. [27] addressed the lack of discrete element parameters for residual film in cotton-field cultivated layers by calibrating residual film parameters based on the Hertz–Mindlin with Bonding model, and established a relatively accurate discrete element model of residual film. Xing et al. [28] calibrated the discrete element parameters of Hainan lateritic red soil using the angle of repose as the response index, and verified the accuracy of the model through a soil-breaking resistance test. Ye et al. [29] took Arundo donax-like stems as the research object, measured their contact parameters as well as shear and compression mechanical properties, and calibrated the parameters of the Hertz–Mindlin with Bonding model using response indices such as the stacking angle, shear force, and compressive failure force. The validation results showed that the maximum deviation between the physical and simulation tests was 4.81%, indicating that the established model could effectively characterize the contact and failure behavior of Arundo donax-like stems. Du et al. [30] established a three-layer discrete element model of tea stems based on the enhanced bonding method, and calibrated the bonding parameters using the maximum shear force as the response index. The relative errors of the tensile and puncture validation tests were no greater than 3%, indicating that the model could accurately characterize the mechanical response characteristics of tea stems. Li et al. [31] established a discrete element model of alfalfa leaves using a flexible thin-shell particle model, and calibrated the model parameters based on leaf sliding angle and cylinder compression tests, combined with Plackett–Burman, steepest ascent, single-factor, and central composite tests. The results showed that the calibrated parameters could effectively characterize the material properties of alfalfa leaves.

In summary, existing studies on discrete element modeling and parameter calibration have mainly focused on single agricultural materials, such as soil, residual film, crop stems and leaves, while studies on the modeling of the multi-component composite structure of whole plug seedlings remain limited. To address the insufficient characterization of the multi-component composite structure of whole plug seedlings in existing studies, this study incorporated the root–substrate composite of the seedling pot and flexible organs, including the leaf, petiole and stem, into the discrete element modeling object, thereby overcoming the limitations of modeling a single agricultural material or local organ. A whole-plant discrete element model of Shanghai bok choy plug seedling was established using a combined method of component-wise modeling and overall reconstruction. According to the typical loading forms of different components, the ultimate compressive failure load of the seedling pot, leaf compression density, ultimate bending failure load of the petiole and ultimate bending failure load of the stem were used as response indices to perform component-wise calibration and validation of the key bonding parameters of the Bonding V2 model. The results can provide a model basis and parameter reference for discrete element simulation of the interaction between Shanghai bok choy plug seedling and key components of automatic transplanting equipment, as well as for equipment design optimization.

## 2. Materials and Methods

### 2.1. Determination of Material Properties of Shanghai Bok Choy Plug Seedling

#### 2.1.1. Test Materials

**Figure 1:**
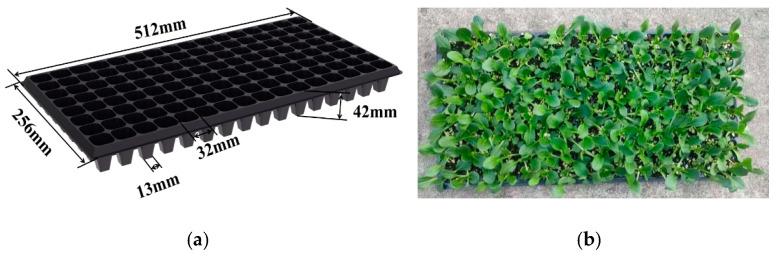
Schematic diagram of 128-cell Shanghai bok choy plug seedling: (**a**) 128-cell plug tray; (**b**) Shanghai bok choy plug seedling.

The tests were conducted in March 2026 at the Key Laboratory of Modern Agricultural Equipment and Technology, Ministry of Education, Jiangsu University. The laboratory temperature was maintained at (20 ± 2) °C, and the relative humidity was (50 ± 5)%. After being transported to the laboratory, the plug seedlings were laid flat on the seedling tray rack and allowed to stand for more than 12 h while maintaining a stable substrate moisture condition, so that the state of the plug seedlings was basically consistent with the testing environment. All tests were carried out under natural daylight conditions during the daytime to reduce the influence of environmental differences on the test results. The test material was 128-cell Shanghai bok choy plug seedlings uniformly cultivated in a seedling nursery factory, with the variety of “Jingguan No. 1”. The tray cells were in the shape of an inverted frustum. The dimensions of the upper and lower end faces were 32 mm × 32 mm and 13 mm × 13 mm, respectively; the cell depth was 42 mm; the included angle β between the two side walls of the cell was 22°; and the center spacing between adjacent cells was 32 mm, as shown in Figure 1. The seedling cultivation period was 30 days. The substrate was prepared from peat, vermiculite, and perlite at a volume ratio of 3:1:1, and the compaction degree after filling was 1.2. Uniform management measures were adopted during seedling cultivation to reduce individual differences among samples. One day before the test, the substrate moisture content was controlled within the range of 60–65%, which is suitable for mechanical transplanting, using the ebb-and-flow irrigation method, to ensure consistency in the state of the test samples [32].

#### 2.1.2. Determination of Morphological Characteristics

**Figure 2:**
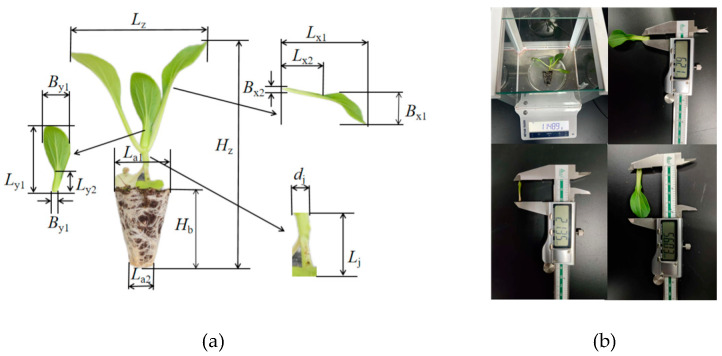
Schematic diagram of morphological parameters and determination test of Shanghai bok choy plug seedling: (**a**) morphological parameters of plug seedling; (**b**) determination of morphological parameters of plug seedling.

Thirty Shanghai bok choy plug seedlings were randomly selected. Since each plug seedling contained two large leaves and two small leaves, the relevant parameters of the large and small leaves were measured separately. Morphological dimensions were measured using a , digital display vernier caliper (Delixi Group Co., Ltd., Zhejiang, China; measuring range: 200 mm; accuracy: 0.01 mm), and mass was measured using an .electronic balance (Mettler Toledo, Zurich, Switzerland; measuring range: 500 g; accuracy: 0.001 g). A total of 30 groups of tests were completed. The measurement process is shown in Figure 2, and the morphological parameter data are listed in Table 1. The coefficients of variation in the morphological parameters of Shanghai bok choy plug seedling were all lower than 10%, indicating that the dispersion of the morphological parameters was small and the morphological parameters were relatively consistent. The samples used in all material property tests were randomly selected from the same cultivation batch. Each plug seedling was used for only one test item. The large and small leaves and the large and small petioles were obtained from the same plug seedling, whereas samples for the other tests were selected separately according to organ type.

#### 2.1.3. Determination of Physical Properties

(1) Determination of Seedling Pot Density

**Figure 3:**
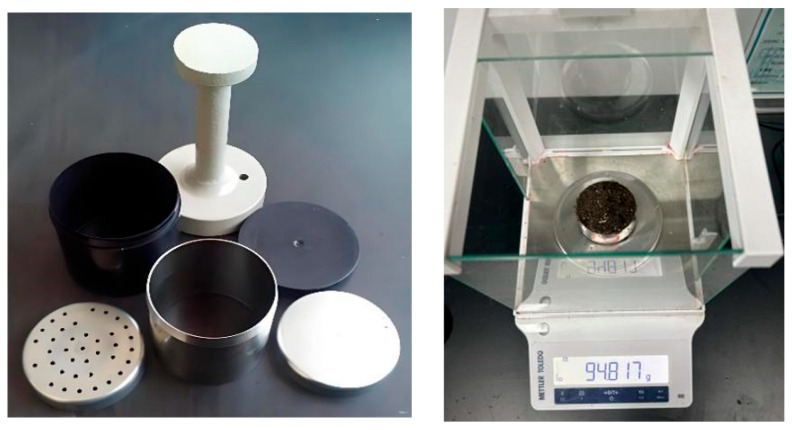
Schematic diagram of seedling pot density determination test.

The seedling pot density was determined using the cutting ring method. Thirty Shanghai bok choy plug seedlings were randomly selected. After separation, the seedling pots were appropriately crushed and mixed uniformly, then placed into the cutting ring. The surface was leveled to ensure that the substrate volume was consistent with the volume of the cutting ring, and the total mass of the cutting ring and substrate was then weighed. The seedling pot density was calculated according to Formula (1). The test was repeated 30 times, as shown in Figure 3.

(1) $$\rho_{j}=\frac{m_{j}}{v_{h}}=\frac{m_{b}-m_{c}}{v_{h}}$$

where *ρ*_j_ is the substrate density, g·cm^−3^; *m*_j_ is the substrate mass, g; *m*_b_ is the total mass of the cutting ring and substrate, g; *m*_c_ is the mass of the cutting ring, g; and *v*_b_ is the volume of the cutting ring, cm^3^.

**Figure 4:**
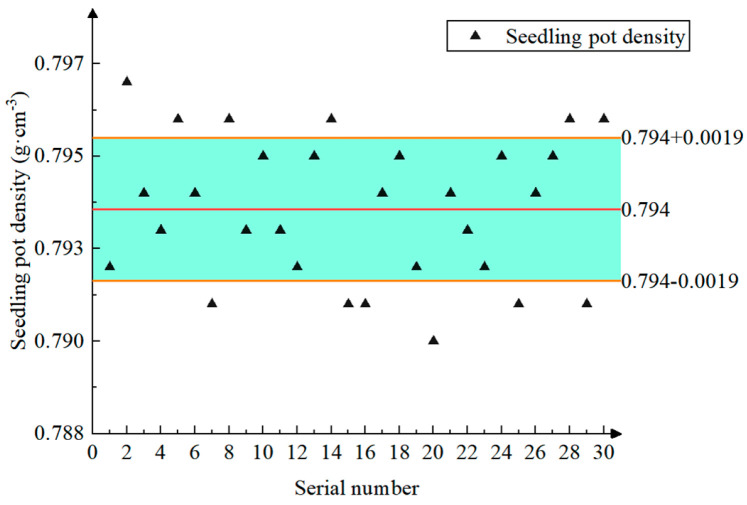
Determination results of seedling pot density.

The determination results of seedling pot density are shown in Figure 4. The mean value was 0.794 g·cm^−3^, the standard deviation was 0.0019 g·cm^−3^, and the coefficient of variation was 0.25%. The results indicated that the seedling pot density data were concentrated and showed good consistency. The differences were mainly attributed to the local compaction degree of the substrate and random errors during sampling and weighing.

(2) Determination of Leaf, Petiole and Stem Density

**Figure 5:**
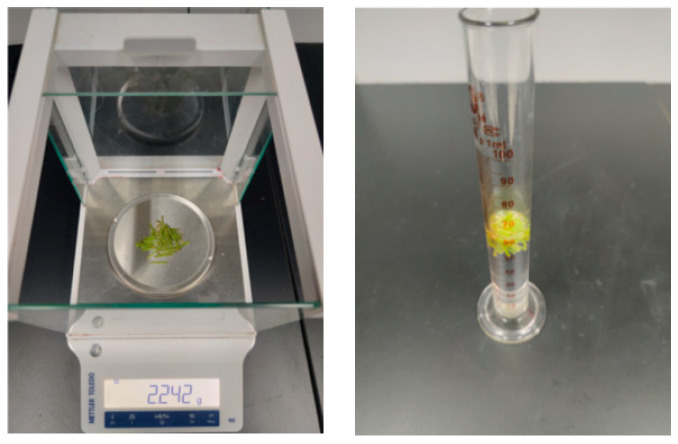
Schematic diagram of density determination test.

The densities of the leaf, petiole and stem were determined using the immersion method. Thirty Shanghai bok choy plug seedlings were randomly selected, and their leaves, petioles and stems were separated. The large and small leaves, as well as the large and small petioles, were sampled separately, with 15 groups measured for each. The leaves were cut into rectangular samples of 20 mm × 20 mm, while the middle sections of the petioles and stems were cut and trimmed into samples approximately 10 mm in length. After the mass of each sample was measured, the sample was completely immersed in a measuring cylinder containing 60 mL of purified water, and the density was calculated according to the displaced water volume. The density was calculated according to Formula (2). The test was repeated 30 times, as shown in Figure 5.

(2) $$\rho_{w}=\frac{m_{w}}{v_{p}}$$

where *ρ*_w_ is the material density, g·cm^−3^; *m*_w_ is the material mass, g; and *v*_p_ is the displaced water volume, mL.

**Figure 6:**
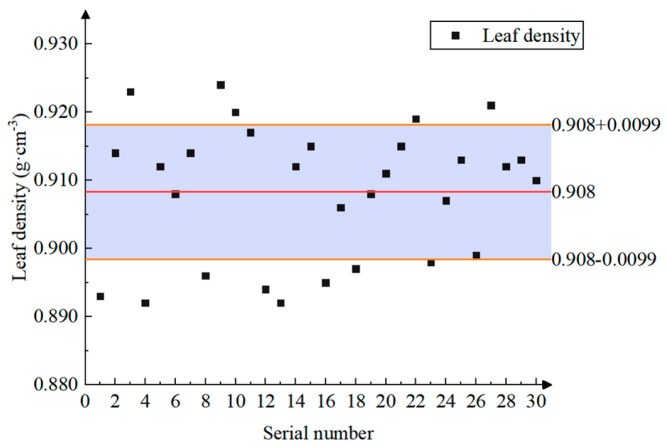
Determination results of leaf density.

**Figure 7:**
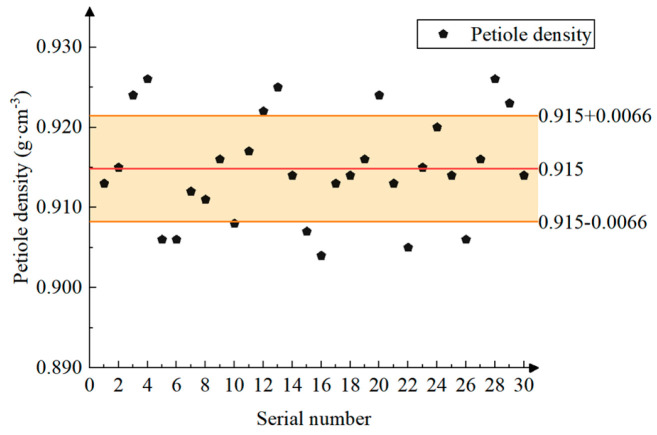
Determination results of petiole density.

**Figure 8:**
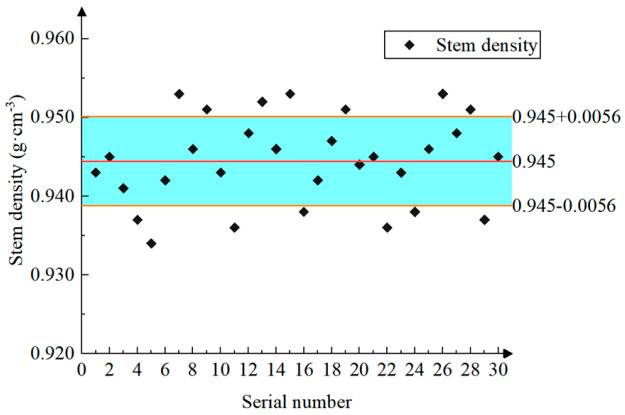
Determination results of stem density.

The determination results of leaf, petiole and stem density are shown in Figure 6, Figure 7 and Figure 8. The mean values were 0.908, 0.915 and 0.945 g·cm^−3^, respectively, and the coefficients of variation were 1.09%, 0.72% and 0.60%, respectively. The results indicated that the density data of each component had low dispersion, concentrated distribution and good sample consistency. Among the 30 groups of data, large and small samples each accounted for 15 groups. The density differences between large and small samples were small, with no significant difference observed, indicating that the size factor had no obvious effect on the densities of leaves and petioles; therefore, unified parameters were used for characterization. Meanwhile, the density followed the order of stem > petiole > leaf, mainly because the stem had a higher vascular bundle content and a denser tissue structure, whereas the petiole and leaf tissues were relatively loose. The data differences were mainly attributed to individual growth differences and reading errors of the measuring cylinder.

#### 2.1.4. Determination of Mechanical Properties

(1) Seedling Pot Flat Plate Compression Test

**Figure 9:**
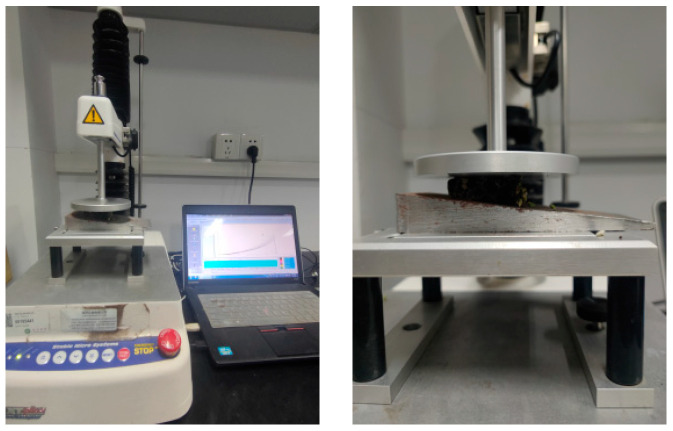
Schematic diagram of seedling pot flat plate compression test.

During transplanting operations, the seedling pot is prone to compressive loads caused by extrusion from the picking needles and collision with working components. Therefore, its compression mechanical properties were determined using a texture analyzer (Stable Micro Systems, UK, model: TA.XTPlus). texture analyzer (TA.XTPlus, Stable Micro Systems Ltd., Godalming, UK).Thirty Shanghai bok choy plug seedlings were randomly selected, and the separated seedling pots were used as samples. An 11° inclined cushion block was adopted to keep the compressed side wall parallel to the loading plate. A P/75 circular flat plate probe was selected, with a loading speed of 1 mm/s and a compression displacement of 10 mm. The test was repeated 30 times, and the load–displacement curves were recorded, as shown in Figure 9.

**Figure 10:**
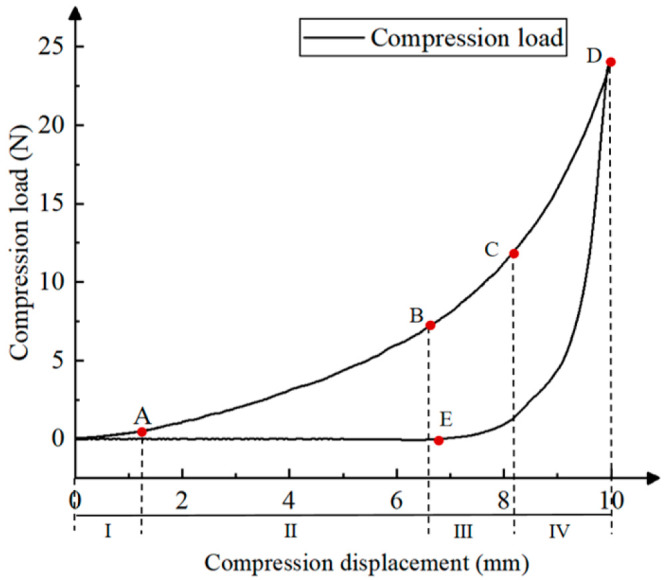
Relationship curve between compression load and displacement of seedling pot.

According to Figure 10, the compression process can be divided into four loading stages and one unloading–rebound stage. Stage I is the elastic deformation stage. Before point A, the internal particle contacts in the seedling pot gradually increased, and the load increased slowly with displacement, while the overall structure remained basically stable. Stage II is the yield softening stage, during which the substrate particles slipped and rearranged, and the pores were gradually compressed. At point B, the seedling pot began to yield and exhibited obvious plastic deformation. Stage III is the bio-compaction hardening stage, during which the substrate particles were further compacted, the interlocking effect between the roots and substrate was enhanced, and the load increased rapidly. Point C was the ultimate failure point. Stage IV is the forced compaction stage. After structural rupture of the seedling pot, the substrate continued to be compacted, and the load increased significantly. Point D was the maximum load point. The D–E segment represents the unloading–rebound stage. After unloading, only slight rebound occurred in the seedling pot, indicating that the compression process was obviously irreversible.

**Figure 11:**
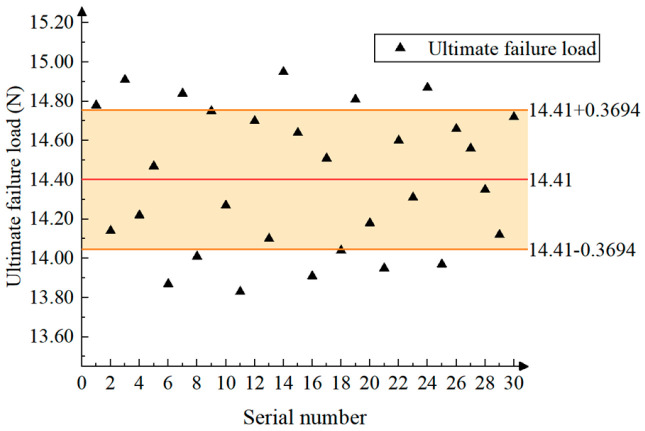
Test results of ultimate compressive failure load of seedling pot.

The ultimate compressive failure load of the seedling pot was obtained from the compression load–displacement curve, and the data are shown in Figure 11. The mean value was 14.41 N, the standard deviation was 0.3694 N, and the coefficient of variation was 2.56%. The results indicated that the ultimate compressive failure load of the seedling pot had low dispersion, and the mechanical response during the compression failure stage was relatively stable.

The approximately linear stage of the force–displacement curve was selected as the elastic deformation region of the seedling pot. Based on Hooke’s law, the elastic modulus of the seedling pot was calculated according to Formula (3), providing a theoretical basis for the establishment and calibration of the discrete element model of the seedling pot.

(3) $$E_{1}=\frac{\Delta F_{1}\cdot l_{1}}{\Delta l_{1}\cdot A_{1}}$$

where *E*_1_ is the elastic modulus of the material, MPa; Δ*F*_1_ is the variation in compression force during the linear change stage of the material, N; Δ*l*_1_ is the variation in compression displacement during the linear change stage of the material, mm; *l*_1_ is the original length of the material, mm; and *A*_1_ is the cross-sectional area of the material, mm^2^.

**Figure 12:**
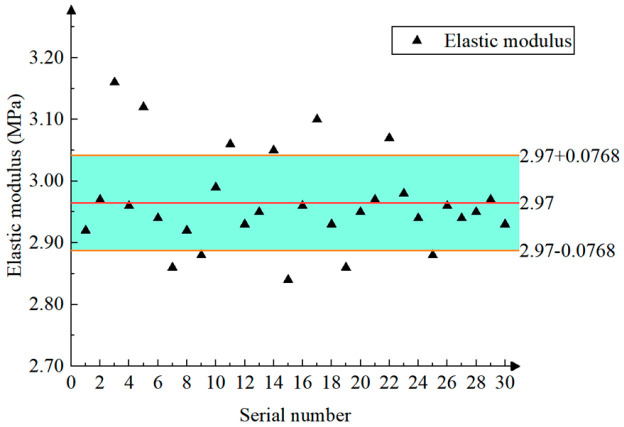
Test results of elastic modulus of seedling pot.

The elastic modulus of the seedling pot was calculated according to Formula (3), and the data are shown in Figure 12. The mean elastic modulus of the seedling pot was 2.97 MPa, the standard deviation was 0.0768 MPa, and the coefficient of variation was 2.60%. The results indicated that the elastic modulus data of the seedling pot had low dispersion and an overall concentrated distribution.

(2) Leaf Tensile Test

**Figure 13:**
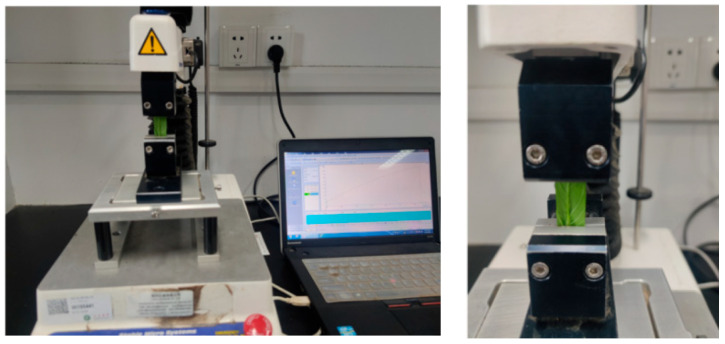
Schematic diagram of leaf tensile mechanical properties test.

Shanghai bok choy leaves are thin, flexible structures with good ductility. During seedling picking, seedling separating and planting, they mainly exhibit bending deformation, and the probability of structural failure is relatively low. Therefore, only the leaf elastic modulus was obtained using a texture analyzer through tensile tests. Thirty plug seedlings were randomly selected, and the large and small leaves were cut along the vein direction into strip samples of 30 mm × 15 mm and 20 mm × 10 mm, respectively. The test was conducted using a vertical uniaxial tensile method, with a loading speed of 1 mm/s and a tensile distance of 5 mm. Data acquisition began when the load reached 0.1 N. The large and small leaves were each tested 30 times, and the load–displacement curves were recorded, as shown in Figure 13.

**Figure 14:**
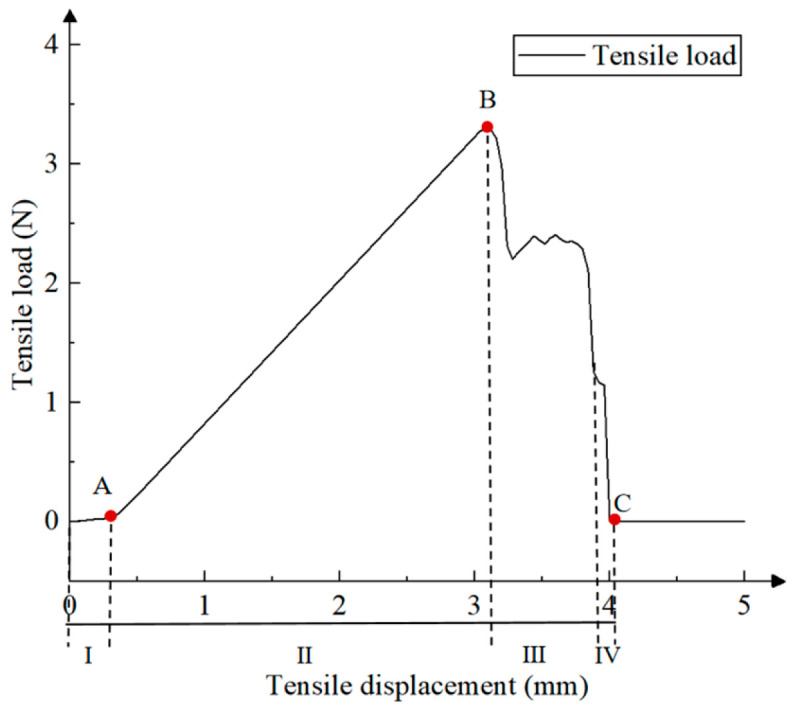
Relationship curve between tensile load and displacement of leaf.

According to Figure 14, the leaf tensile process can be divided into four stages. Stage I is the initial adjustment stage. Before point A, the leaf was gradually straightened, and the load increased slowly. Stage II is the elastic deformation stage, in which the load increased approximately linearly with displacement, and the leaf was in a stable stress state. Point B was the ultimate load failure point. Stage III is the local damage propagation stage. After point B, the load decreased rapidly with fluctuations, indicating that local tissues or veins began to tear, while the leaf still retained a certain residual load-bearing capacity. Stage IV is the complete fracture stage. As the displacement continued to increase, the remaining load-bearing tissues gradually failed. At point C, the leaf was completely fractured, and the load approached zero.

**Figure 15:**
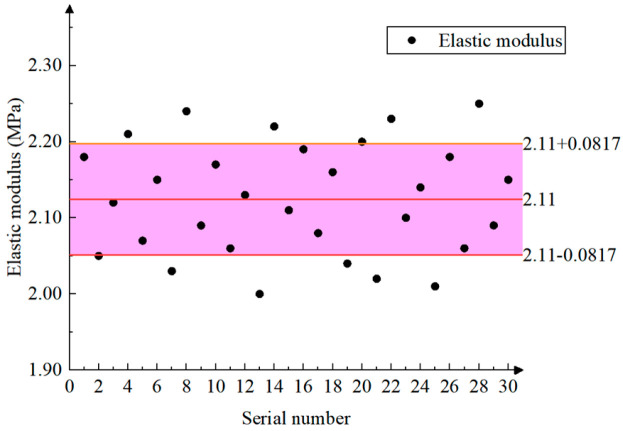
Test results of elastic modulus of large leaf.

**Figure 16:**
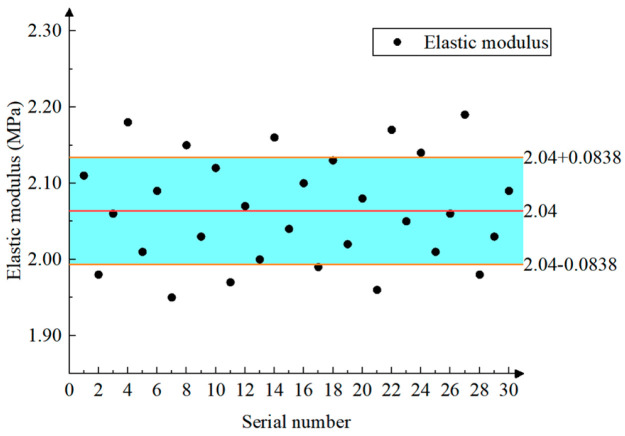
Test results of elastic modulus of small leaf.

The elastic moduli of the large and small leaves were calculated according to Formula (3), and the data are shown in Figure 15 and Figure 16. The mean elastic moduli of the large and small leaves were 2.11 MPa and 2.04 MPa, respectively; the standard deviations were 0.0817 MPa and 0.0838 MPa, respectively; and the coefficients of variation were 3.87% and 4.11%, respectively. The data for both leaves showed low dispersion, and the relative difference was only 3.43%, indicating that their mechanical properties were relatively close. Therefore, the mean value of 2.08 MPa was used to characterize the leaf elastic modulus in the subsequent discrete element modeling.

(3) Leaf Cylinder Compression Test

**Figure 17:**
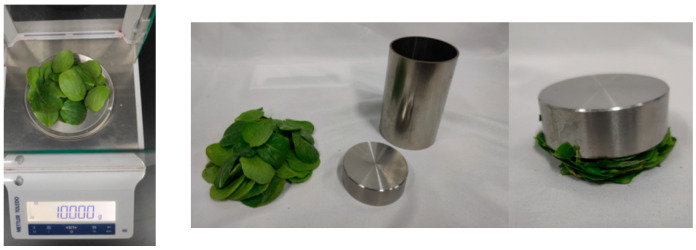
Schematic diagram of leaf cylinder compression test.

Thirty Shanghai bok choy plug seedlings were randomly selected for the leaf cylinder compression test to determine the leaf compression density. The separated large and small leaves were weighed to 10 g, respectively, and naturally stacked in a stainless-steel cylinder with a radius of 25 mm and a height of 100 mm. A cylindrical compression block with a mass of 500 g and a radius of 24.5 mm was then placed on the leaves for compression. After the leaf stack became stable, the distance between the bottom surface of the compression block and the table was measured to determine the compressed volume, and the compression density was calculated according to Formula (4). The test was repeated 30 times, as shown in Figure 17.

(4) $$\rho_{y}=\frac{m_{y}}{v_{y}}=\frac{m_{y}}{s_{y}\times h_{y}}$$

where *ρ*_y_ is the leaf compression density, g·cm^−3^; *m*_y_ is the leaf mass, g; *s*_y_ is the cross-sectional area of the cylinder, mm^2^; and *h*_y_ is the distance between the bottom surface of the compression block and the table after compression, mm.

**Figure 18:**
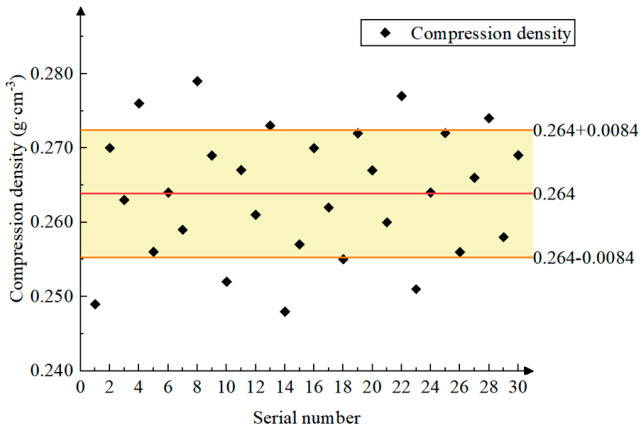
Test results of compression density of large leaf.

**Figure 19:**
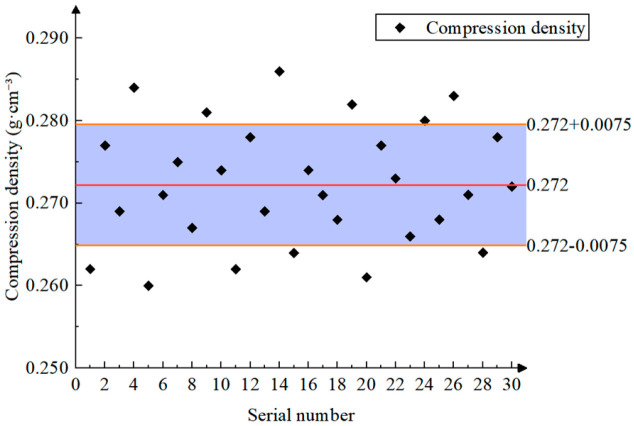
Test results of compression density of small leaf.

The leaf compression density was calculated according to Formula (4), and the data are shown in Figure 18 and Figure 19. The mean compression densities of the large and small leaves were 0.264 g·cm^−3^ and 0.272 g·cm^−3^, respectively; the standard deviations were 0.0084 g·cm^−3^ and 0.0075 g·cm^−3^, respectively; and the coefficients of variation were 3.20% and 2.77%, respectively. The data for both leaves showed low dispersion, and the relative difference was only 2.94%, indicating that their compression and stacking characteristics were relatively close. Therefore, the mean value of 0.268 g·cm^−3^ was used to characterize the leaf compression density in the subsequent discrete element modeling.

(4) Petiole Three-Point Bending Test

**Figure 20:**
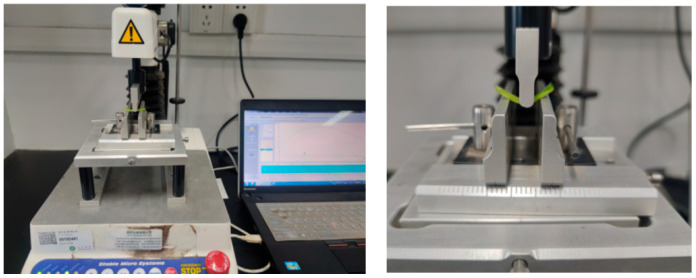
Schematic diagram of petiole three-point bending test.

The petiole is a slender, flexible structure and is prone to bending deformation during seedling picking, seedling separating and planting. Therefore, its bending mechanical properties were determined using a texture analyzer. Thirty plug seedlings were randomly selected, and 25 mm large petiole segments and 20 mm small petiole segments were cut for the three-point bending test. The test span was 10 mm, the loading speed was 1 mm/s, and the loading displacement was 4 mm. Data acquisition began when the load reached 0.1 N. The large and small petioles were each tested 30 times, and the load–displacement curves were recorded, as shown in Figure 20.

**Figure 21:**
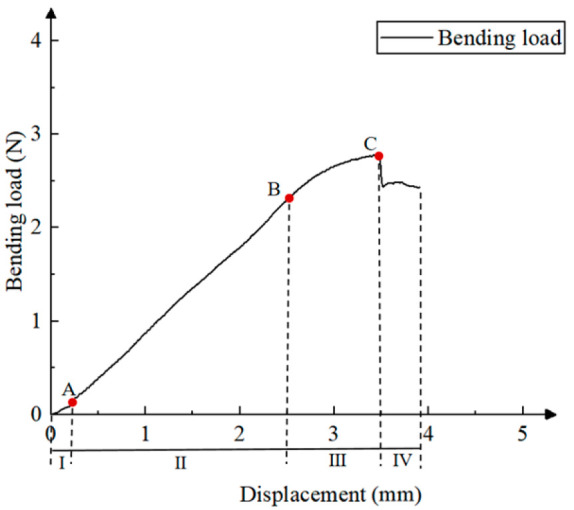
Relationship curve between bending load and displacement of petiole.

According to Figure 21, the petiole bending process can be divided into four stages. Stage I is the contact adjustment stage. Before point A, the petiole gradually contacted the probe and support points, and the load increased slowly. Stage II is the elastic deformation stage, in which the load increased approximately linearly with displacement, and point B was the yield point. Stage III is the elastoplastic deformation stage, during which the curve slope decreased and the petiole gradually underwent plastic deformation. Point C was the ultimate failure point. Stage IV is the damage propagation and load-bearing attenuation stage. After point C, the load decreased with slight fluctuations, indicating that the petiole was damaged, while it still retained a certain residual load-bearing capacity.

**Figure 22:**
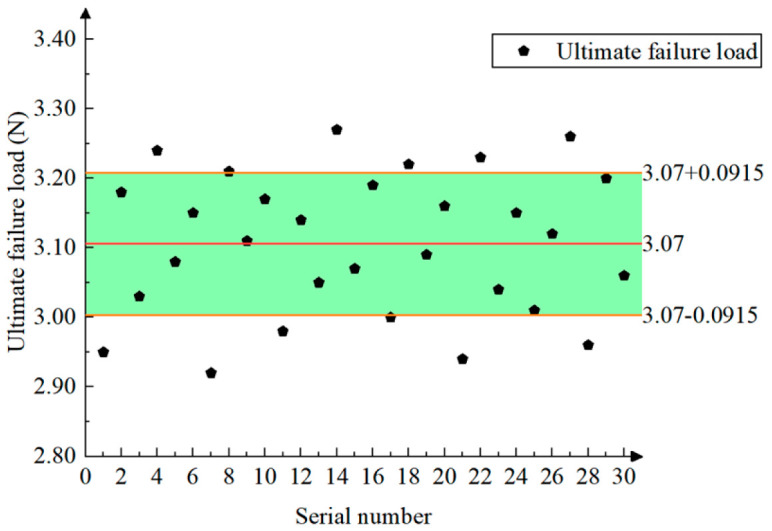
Test results of ultimate bending failure load of large petiole.

**Figure 23:**
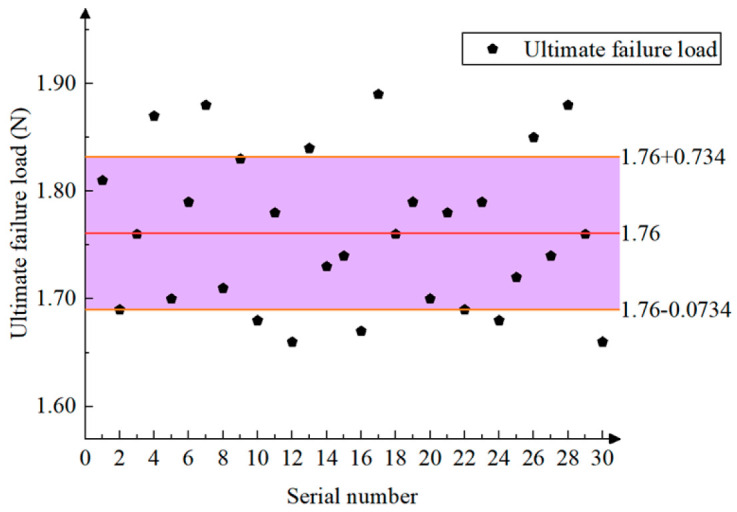
Test results of ultimate bending failure load of small petiole.

The ultimate bending failure load of the petiole was obtained from the bending load–displacement curve, and the results are shown in Figure 22 and Figure 23. The mean ultimate failure loads of the large and small petioles were 3.07 N and 1.76 N, respectively; the standard deviations were 0.0915 N and 0.0734 N, respectively; and the coefficients of variation were 2.98% and 4.16%, respectively. The results indicated that the ultimate failure load data of both petioles had low dispersion, and the mechanical response during the bending failure stage was relatively stable. The ultimate failure load of the large petiole was higher than that of the small petiole, indicating that it had stronger load-bearing capacity and failure resistance.

The approximately linear stage of the bending force–displacement curve was selected as the elastic deformation region of the petiole. Based on the three-point bending theory, the bending elastic modulus of the petiole was calculated according to Formula (5).

(5) $$E_{2}=\frac{4L_{k}^{3}\cdot \Delta F_{2}}{3\pi a_{2}{b_{2}}^{3}\cdot \Delta l_{2}}$$

where *E*_2_ is the bending elastic modulus of the petiole, MPa; Δ*F*_2_ is the variation in bending force during the linear change stage of the petiole, N; Δ*l*_2_ is the variation in bending displacement during the linear change stage of the petiole, mm; *L*_k_ is the span length, mm; *a*_2_ is the major-axis diameter of the elliptical cross-section, mm; and *b*_2_ is the minor-axis diameter of the elliptical cross-section, mm.

**Figure 24:**
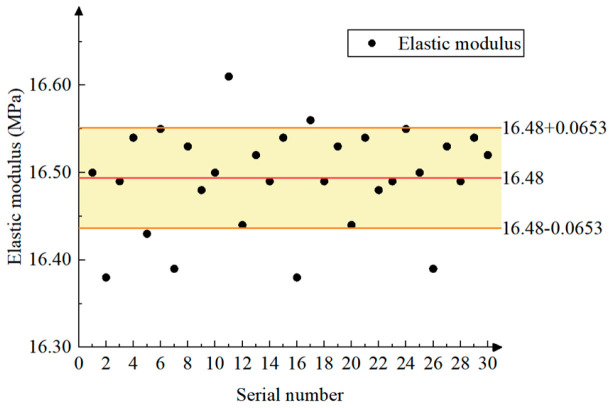
Test results of elastic modulus of large petiole.

**Figure 25:**
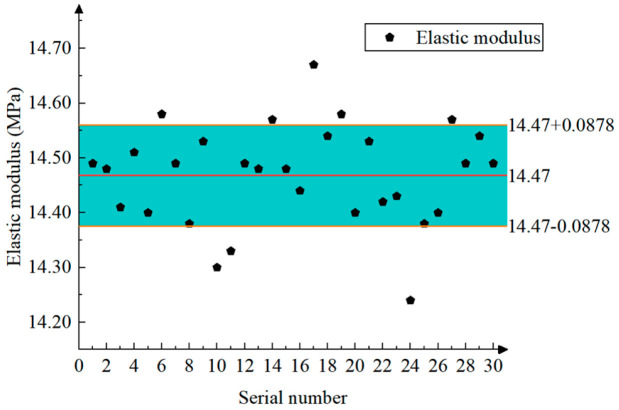
Test results of elastic modulus of small petiole.

The elastic moduli of the large and small petioles were calculated according to Formula (5), and the data are shown in Figure 24 and Figure 25. The mean elastic moduli of the large and small petioles were 16.48 MPa and 14.47 MPa, respectively, and the coefficients of variation were 0.40% and 0.61%, respectively. The data for both petioles showed low dispersion. The elastic modulus of the large petiole was higher than that of the small petiole, indicating that it had stronger resistance to elastic deformation, which was mainly related to its more developed vascular tissue and denser structure.

(5) Stem Three-Point Bending Test

**Figure 26:**
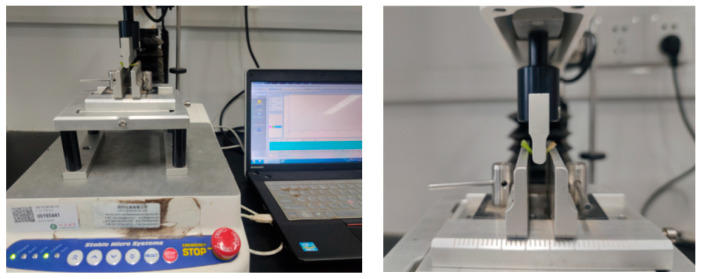
Schematic diagram of stem three-point bending test.

The stem is the main load-bearing structure connecting the seedling pot and leaves, and is prone to bending deformation during seedling picking, seedling separating and planting. Therefore, its bending mechanical properties were determined using a texture analyzer. Thirty plug seedlings were randomly selected, and stem segments with a length of 20 mm, complete morphology and uniform thickness were cut for the three-point bending test. The test span was 10 mm, the loading direction was perpendicular to the stem axis, the loading speed was 1 mm/s, and the loading displacement was 5 mm. Data acquisition began when the load reached 0.1 N. The test was repeated 30 times, and the load–displacement curves were recorded, as shown in Figure 26.

**Figure 27:**
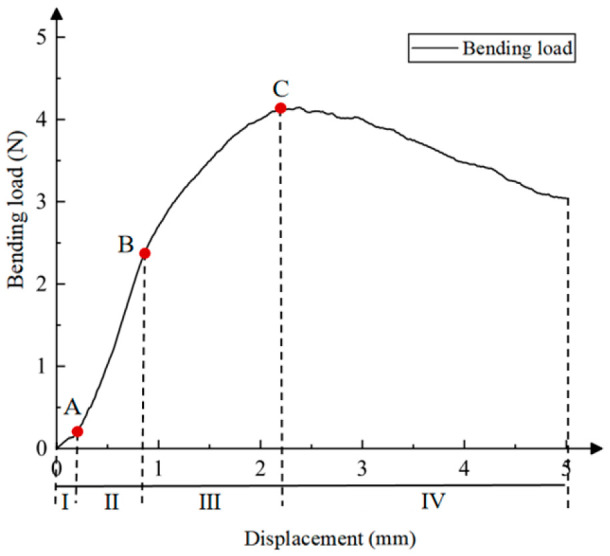
Relationship curve between bending load and displacement of stem.

According to Figure 27, the stem bending process can be divided into four stages. Stage I is the contact adjustment stage. Before point A, the stem gradually contacted the probe and support points, and the load increased slowly. Stage II is the elastic deformation stage. In the A–B segment, the load increased rapidly, and the internal tissues of the stem jointly bore the load. Point B was the yield point. Stage III is the elastoplastic deformation stage. In the B–C segment, the curve slope decreased, and the stem gradually underwent plastic deformation. Point C was the ultimate failure point. Stage IV is the damage propagation and load-bearing attenuation stage. After point C, the load decreased with fluctuations, indicating that the internal tissues were gradually damaged, while the stem still retained a certain residual load-bearing capacity.

**Figure 28:**
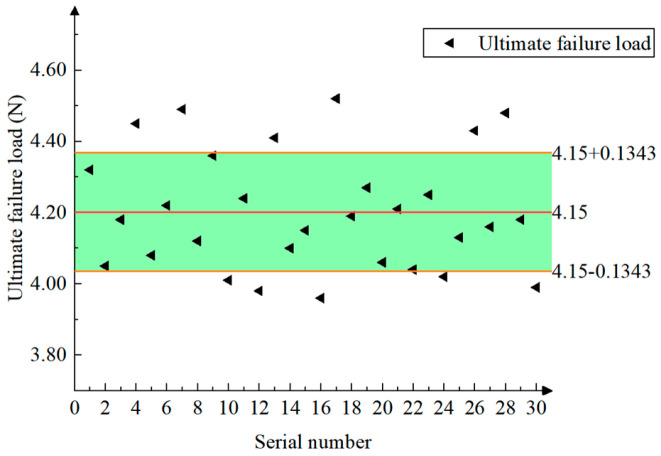
Test results of ultimate bending failure load of stem.

The ultimate bending failure load of the stem was obtained from the stem bending load–displacement curve, and the data are shown in Figure 28. The mean value was 4.15 N, the standard deviation was 0.1343 N, and the coefficient of variation was 3.24%. The results indicated that the ultimate bending failure load of the stem had low dispersion, and the mechanical response during the bending failure stage was relatively stable.

The approximately linear stage of the bending force–displacement curve was selected as the elastic deformation region of the stem. Based on the three-point bending theory, the bending elastic modulus of the stem was calculated according to Formula (6).

(6) $$E_{3}=\frac{4L_{k}^{3}\cdot \Delta F_{3}}{3\pi d^{4}\cdot \Delta l_{3}}$$

where *E*_3_ is the bending elastic modulus of the stem, MPa; Δ*F*_3_ is the variation in bending force during the linear change stage of the stem, N; Δ*l*_3_ is the variation in bending displacement during the linear change stage of the stem, mm; *L*_k_ is the span length, mm; and *d* is the stem diameter, mm.

**Figure 29:**
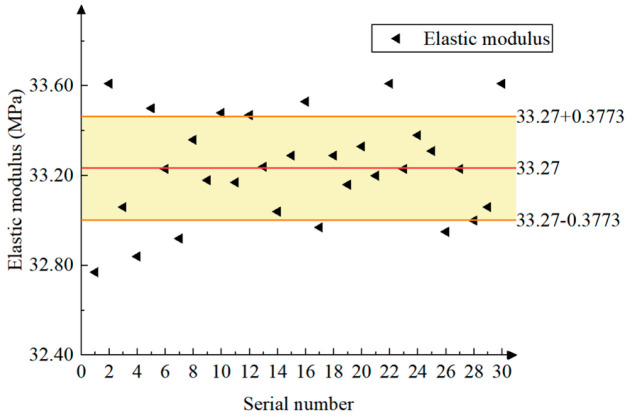
Test results of elastic modulus of stem.

The elastic modulus of the stem was calculated according to Formula (6), and the data are shown in Figure 29. The mean elastic modulus of the stem was 33.27 MPa, the standard deviation was 0.3773 MPa, and the coefficient of variation was 1.13%, indicating that the data had low dispersion and a concentrated distribution.

#### 2.1.5. Determination of Contact Properties

(1) Determination of Static Friction Coefficient

**Figure 30:**
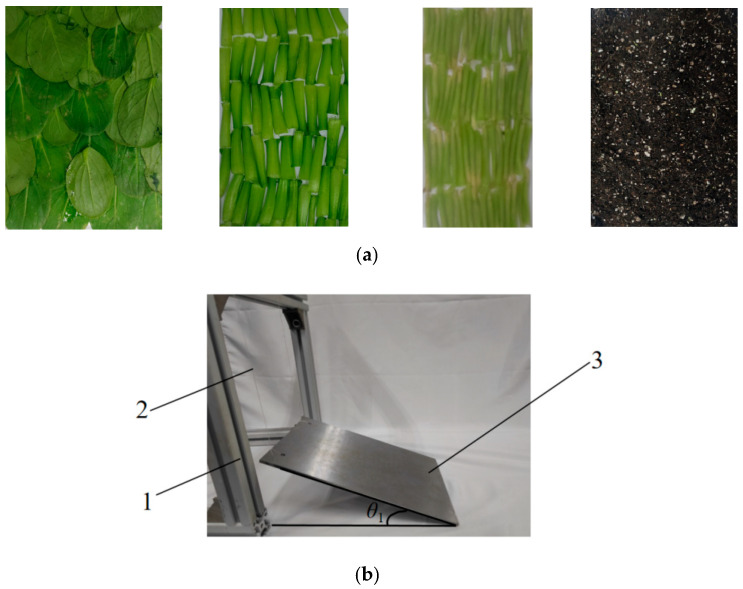
Schematic diagram of static friction coefficient determination test: (**a**) panels made of leaf, petiole, stem and seedling pot; (**b**) static friction coefficient test device: 1. inelastic fishing line; 2. base frame; 3. stainless steel plate.

According to the actual contact relationship between the plug seedling and machine during transplanting, a self-made inclined-plane friction test device was used to determine the static friction coefficients of different components of Shanghai bok choy plug seedling and those between the components and contact materials. The test objects included seedling pot–steel plate, seedling pot–plastic plate, seedling pot–seedling pot, leaf–steel plate, leaf–leaf, petiole–steel plate, petiole–petiole, stem–steel plate and stem–stem combinations. Before determining the static friction coefficients between materials of the same type, the substrate with roots, leaf, petiole and stem were fixed on the surface of a flat plate to prepare the corresponding material panels. The static friction coefficient was determined using the inclined-plane method. Taking the seedling pot–steel plate combination as an example, the seedling pot was placed vertically at the center of the steel plate, and the inclined plane was slowly lifted. When the seedling pot was about to slide, the critical inclination angle of the inclined plane was measured using a digital angle gauge (measuring range: 360°, accuracy: 0.1°). The other contact combinations were determined using the same method, and each group was repeated 30 times. The test is shown in Figure 30, and the static friction coefficient was calculated according to Formula (7).

(7) $$\mu_{\mathrm{sw}}=\tan\theta_{1}$$

where *μ*_sw_ is the static friction coefficient of the material, and *θ*_1_ is the critical angle between the inclined plane and the horizontal direction when the sample begins to slide, °.

According to the static friction coefficient test results, the static friction coefficients between the seedling pot, leaf, petiole and stem and different contact materials were calculated, respectively. The relevant data are shown in Table 2.

(3) Determination of Dynamic Friction Coefficient

**Figure 31:**
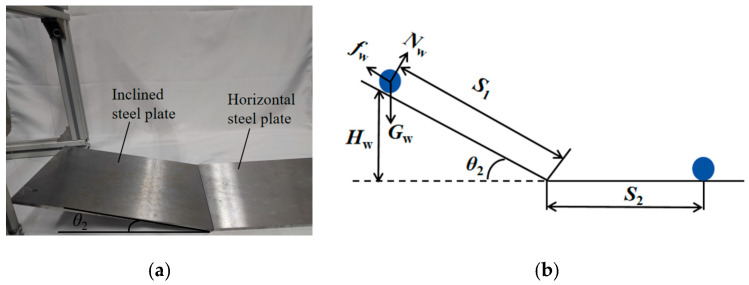
Schematic diagram of dynamic friction coefficient test device and principle: (**a**) dynamic friction coefficient test device; (**b**) principle of dynamic friction coefficient test.

Before the test, the seedling pot and leaf were made into approximate spheres with a diameter of about 15 mm, and the petiole was trimmed into a cylinder with a diameter of about 3 mm to reduce the influence of shape differences on the motion state. Taking the seedling pot–steel plate combination as an example, a horizontal steel plate was connected to the bottom of the inclined steel plate. After release, the seedling pot rolled along the inclined plane and finally stopped on the horizontal plate. The steel plate inclination angle, release height, inclined-plane motion distance and horizontal rolling distance were measured, respectively. The other contact combinations were determined using the same method, and each group was repeated 30 times. The test and principle are shown in Figure 31a and Figure 31b, respectively.

According to the energy variation in the seedling pot sphere on the inclined and horizontal sections, combined with the principle of energy conservation, the dynamic friction coefficient between the seedling pot and steel plate can be calculated using Formulas (8) and (9).

(8) $$G_{w}H_{w}=\mu_{\mathrm{dw}}G_{w}\left(S_{1}\cos\theta_{2}+S_{2}\right)$$

(9) $$\mu_{\mathrm{dw}}=\frac{H_{w}}{S_{1}\cos\theta_{2}+S_{2}}$$

where *G*_w_ is the gravity of the material, N; *H*_w_ is the release height of the material, mm; *μ*_dw_ is the dynamic friction coefficient of the material; *S*_1_ is the rolling distance of the material on the inclined plane, mm; *θ*_2_ is the angle between the inclined plane and the horizontal plane, °; and *S*_2_ is the rolling distance of the material on the horizontal plane, mm.

According to the dynamic friction coefficient test results, the dynamic friction coefficients between the seedling pot, leaf, petiole and stem and different contact materials were calculated, respectively. The relevant data are shown in Table 3.

(5) Determination of Collision Restitution Coefficient

**Figure 32:**
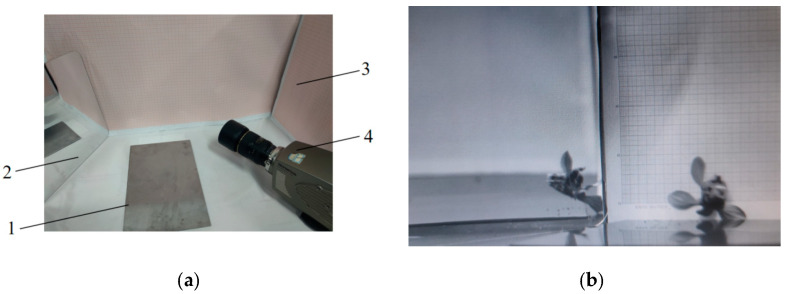
Schematic diagram of collision restitution coefficient determination test: (**a**) collision restitution coefficient test device: 1. steel plate; 2. mirror; 3. coordinate paper; 4. high-speed camera; (**b**) high-speed camera image.

Before the test, the materials were standardized. The seedling pot retained its original morphology, while the leaf, petiole and stem were cut to uniform sizes to reduce the influence of sample differences on the collision results. A self-made device and the free-fall collision method were used to determine the collision restitution coefficient. A high-speed camera (Olympus, Japan, model: i-SPEED TR) was fixed horizontally, and a reflector positioned at 135° to the reference wall was used to synchronously record the motion trajectories of the sample before and after collision. A 1 mm coordinate grid paper was attached to the reference wall for displacement calibration. The test is shown in Figure 32, and the collision restitution coefficient was calculated according to Formula (10).

(10) $$e_{w}=\frac{v_{\mathrm{tw}}}{v_{0w}}$$

where *e*_w_ is the collision restitution coefficient of the material; *v*_tw_ is the instantaneous rebound velocity of the material after collision, m·s^−1^; and *v*_0w_ is the instantaneous velocity of the material before collision, m·s^−1^.

Taking the determination of the collision restitution coefficient between the seedling pot and steel plate as an example, a spatial rectangular coordinate system XYZ was established. The coordinates at the moment of collision were set as (*x*_1_, *z*_1_), and the mirror coordinates were (*y*_1_, *z*_1_). The geometric center coordinates of the seedling pot k frames before collision were (*x*_0_, *z*_k_), and the mirror coordinates were (*y*_1_, *z*_1_). The seedling pot underwent uniformly accelerated motion in the vertical direction; therefore, the instantaneous velocity of the seedling pot before collision with the steel plate is expressed by Formula (11).

(11) $$v_{0w}=\frac{z_{k}-z_{1}}{t_{k}}+\frac{1}{2}gt_{k}$$

where *t*_k_ is the time for the material to move through *k* frames.

After the seedling pot rebounded and moved for *j* frames, the motion time of the seedling pot was set as *t*_j_, the coordinates were (*x*_j_, *z*_j_), and the mirror coordinates were (*y*_j_, *z*_j_). The seedling pot underwent uniformly decelerated motion with an acceleration of *g* in the vertical direction. Since the instantaneous velocity at the intermediate moment during rebound was equal to the average velocity within this period, the upward instantaneous motion velocity of the seedling pot after rebound is expressed by Formulas (12) and (13).

(12) $$\left\{\begin{array}{l} v_{\mathrm{xm}}=\frac{x_{j}-x_{1}}{t_{k}}\\ v_{\mathrm{ym}}=\frac{y_{j}-y_{1}}{t_{k}}\\ v_{\mathrm{zm}}=\frac{z_{j}-z_{1}}{t_{k}}+\frac{1}{2}gt_{k} \end{array}\right.$$

(13) $$v_{\mathrm{tw}}=\sqrt{v_{\mathrm{xm}}^{2}+v_{\mathrm{ym}}^{2}+v_{\mathrm{zm}}^{2}}$$

where *v*_xm_ is the instantaneous velocity in the *X*-axis direction, m·s^−1^; *v*_ym_ is the instantaneous velocity in the *Y*-axis direction, m·s^−1^; and *v*_zm_ is the instantaneous velocity in the *Z*-axis direction, m·s^−1^.

Substituting Formulas (11)–(13) into Formula (10), the collision restitution coefficient of the seedling pot is expressed by Formula (14).

(14) $$e=\frac{2t_{k}\sqrt{\frac{{\left(x_{j}-x_{1}\right)}^{2}+{\left(y_{j}-y_{1}\right)}^{2}+{\left(z_{j}-z_{1}\right)}^{2}}{t_{j}^{2}}}}{2\left(z_{k}-z_{1}\right)+gt_{m}^{2}}$$

The collision restitution coefficients of the other combinations were determined using the same method, with the corresponding collision test materials replaced according to different combinations. Each group of tests was repeated 30 times. According to the collision restitution coefficient test results, the collision restitution coefficients between the seedling pot, leaf, petiole and stem of Shanghai bok choy plug seedling and different contact materials were calculated, respectively. The relevant data are shown in Table 4.

## 3. Establishment of Discrete Element Model of Shanghai Bok Choy Plug Seedling

Because the seedling pot, leaf, petiole and stem differ in material composition, structural morphology and mechanical properties, a combined method of component-wise modeling and overall reconstruction was used to establish the discrete element model of Shanghai bok choy plug seedling. During modeling, the seedling pot, leaf, petiole and stem were filled with particles separately to obtain the spatial coordinates of each particle. These particles were then assembled in a unified coordinate system to form the meta-particle set of the whole Shanghai bok choy plug seedling, thereby completing the establishment of its discrete element model.

### 3.1. Establishment of Discrete Element Model of Seedling Pot

**Figure 33:**
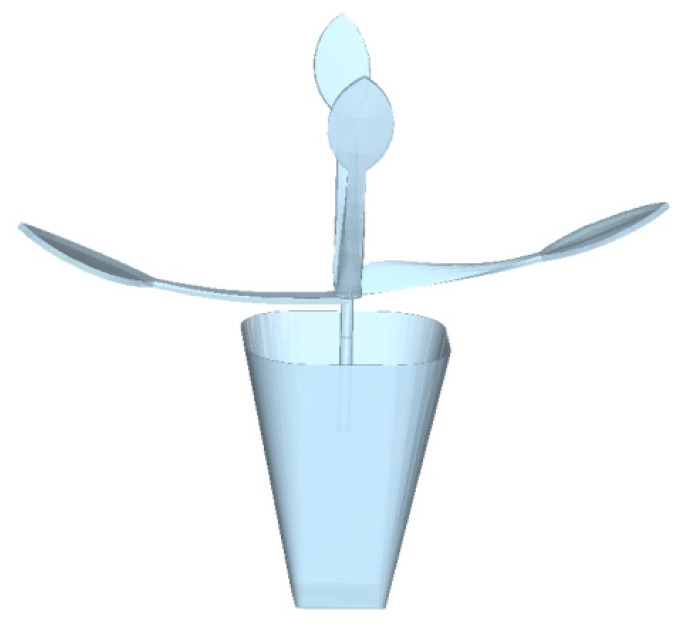
Schematic diagram of three-dimensional model of Shanghai bok choy plug seedling.

Based on the morphological parameters of Shanghai bok choy plug seedling obtained in Section 2.1, a three-dimensional geometric model of Shanghai bok choy plug seedling was established at a 1:1 scale using SolidWorks 2016 software. The model was imported into EDEM 2024 software in STEP format and defined as a solid entity, providing a basis for subsequent particle filling and discrete element modeling, as shown in Figure 33.

**Figure 34:**
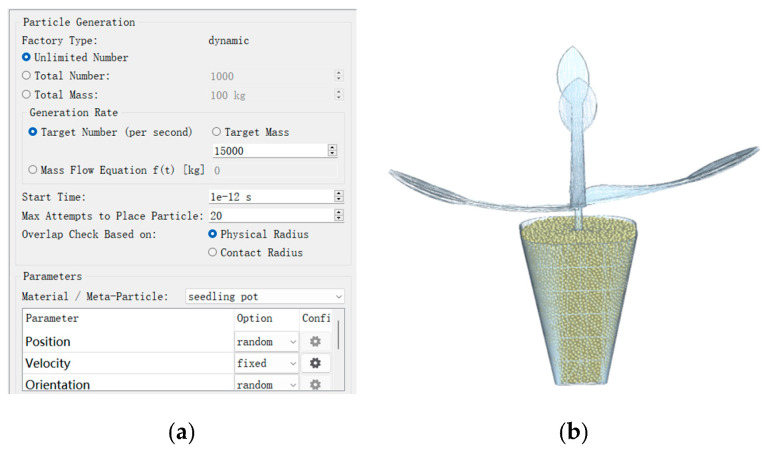
Schematic diagram of particle filling for the seedling pot discrete element model: (**a**) particle factory setting; (**b**) discrete element model of seedling pot.

In the Bulk Material module of EDEM 2024 software, the seedling pot particle material was added and named seedling pot. The material properties of the seedling pot were set according to the relevant physical parameters measured previously. The seedling pot was modeled using single-sphere particles, with a physical radius of 0.5 mm and a contact radius 1.2–1.3 times the physical radius. To achieve particle filling, a virtual plane and particle factory were set at the upper end face of the tray cell. The particles were randomly and continuously generated at a rate of 15,000 particles/s, and then fell, collided and accumulated under gravity until the seedling pot region was filled to form the initial particle model. Since the three-dimensional geometric model was defined as a solid entity, the particles could only be distributed within the effective filling region corresponding to the seedling pot. In addition, because the spatial region of the stem had been preoccupied by the solid geometry, particles could not enter this region. Therefore, the generated seedling pot particles did not overlap with the spatial coordinates of the subsequent stem discrete spherical elements. The seedling pot particle distribution obtained using this method not only met the requirements for establishing the meta-particle model but also provided a basis for the subsequent export of particle center coordinates and coordinate splicing of the whole-plant discrete element model of the plug seedling, as shown in Figure 34.

**Figure 35:**
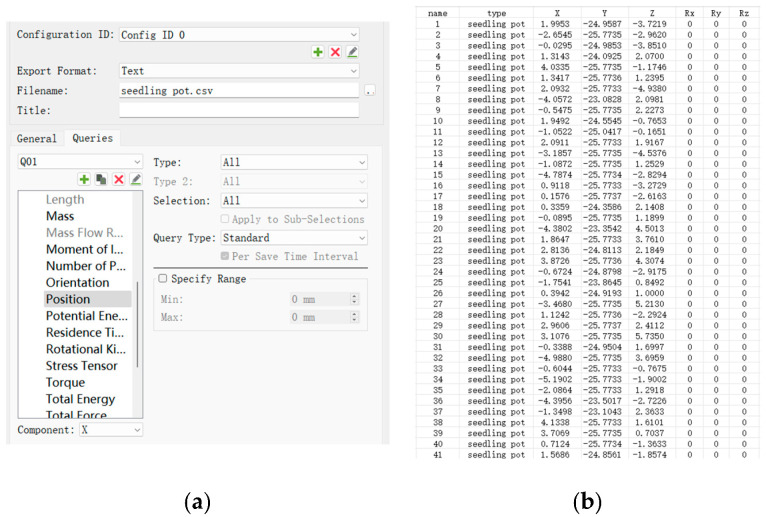
Schematic diagram of seedling pot particle coordinate data export: (**a**) exporting particle coordinate data; (**b**) Excel file of particle coordinate data.

After particle filling was completed and the particle system became stable, the position data of the simulation results were exported as an Excel file. The spatial position coordinates of all particles inside the seedling pot at the end of the simulation, namely the center coordinates of each particle, were extracted. This coordinate information could effectively characterize the spatial distribution, morphology and accumulation features of the particles after seedling pot filling. Based on the exported position coordinates of the seedling pot particles, a basis was provided for subsequent unified splicing and assembly with the particle coordinates of structures such as the stem, petiole and leaf, thereby completing the establishment of the meta-particle model of the whole Shanghai bok choy plug seedling, as shown in Figure 35.

### 3.2. Establishment of Discrete Element Models of Leaf Petiole and Stem

**Figure 36:**
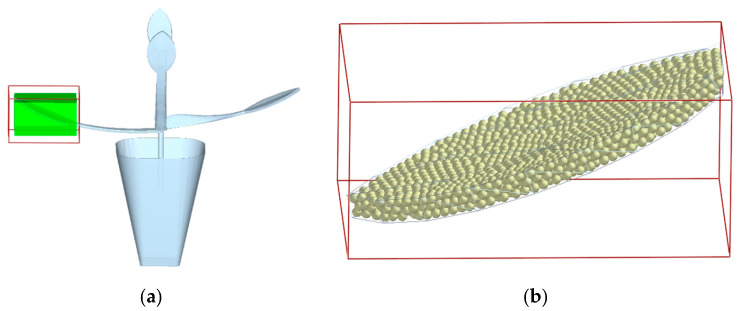
Schematic diagram of particle filling for the leaf discrete element model: (**a**) adjustment of computational domain; (**b**) leaf particle filling.

In the Bulk Material module of EDEM 2024 software, the leaf particle material was added and named leaf. The material parameters of the leaf were set according to the physical parameters measured previously. The leaf was modeled using single-sphere particles, with a physical radius of 2 mm. The three-dimensional leaf model was defined as a virtual body, and a virtual cuboid particle factory matching the leaf size was established by adjusting the computational domain to achieve particle filling in the corresponding leaf region. The setting of the particle factory was the same as that used for seedling pot particle generation. After particle filling was completed, the leaf geometric model was restored as a solid entity, and the center coordinate data of all particles were exported to generate an Excel file. These data served as the basis for the subsequent generation of the leaf meta-particle model and assembly of the whole-plant discrete element model of Shanghai bok choy plug seedling, as shown in Figure 36. The modeling and coordinate export methods for the petiole and stem were the same as those for the leaf, with only the computational domain and particle factory size adjusted.

### 3.3. Generation of Meta-Particles of Shanghai Bok Choy Plug Seedling

**Figure 37:**
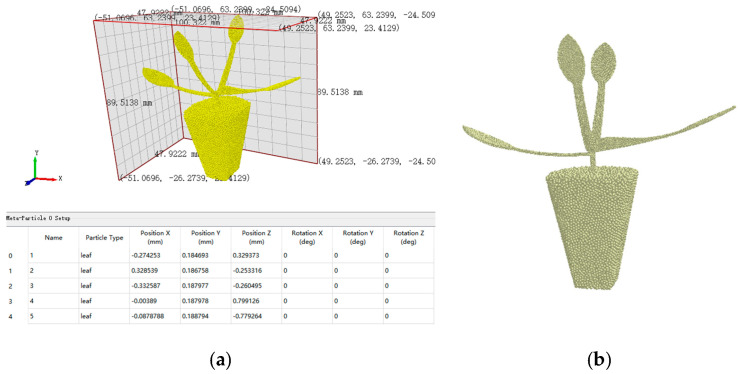
Schematic diagram of meta-particle generation and discrete element model establishment: (**a**) generation of meta-particle; (**b**) establishment of discrete element model.

After particle filling of the seedling pot, leaf, petiole and stem were completed, the coordinate data of particles in each component were exported and corresponding Excel files were generated. The coordinate data were organized and spliced in a unified coordinate system to form the meta-particle coordinate set of the whole Shanghai bok choy plug seedling. All combined particle coordinates were imported into the seedling Setup interface of EDEM 2024 software, and a particle factory was created. The particle material was set as the meta-particle seedling, and the generation number was set to 1, thereby completing the establishment of the whole-plant discrete element model of Shanghai bok choy plug seedling, as shown in Figure 37.

### 3.4. Particle Contact Model

Shanghai bok choy plug seedling is composed of multiple components, including the root–substrate composite of the seedling pot, leaf, petiole and stem. These components involve interparticle contact, friction and collision, while also exhibiting continuous deformation and local failure characteristics. Therefore, the contact model should be able to characterize both basic contact behavior and bonding failure behavior. The JKR model is mainly used to describe adhesion between particles and is more suitable for wet cohesive particles or particle systems dominated by surface adhesion, but it is difficult to characterize the continuous deformation and fracture failure of flexible organs such as the leaf, petiole and stem. Although the Hertz–Mindlin with Bonding model can describe interparticle bonding and failure processes, for multi-organ heterogeneous materials such as Shanghai bok choy plug seedling, it is still necessary to further reflect the differences in bonding stiffness, bonding strength and contact range among different components. The Bonding V2 model can characterize the connection strength and failure characteristics of different organs in a component-wise manner through parameters such as normal stiffness per unit area, tangential stiffness per unit area, critical normal stress, critical tangential stress and contact radius. Therefore, in this study, the combined Hertz–Mindlin (no slip) and Bonding V2 contact models were selected to construct the whole-plant discrete element model of Shanghai bok choy plug seedling. The Hertz–Mindlin (no slip) model was used to characterize the basic contact behavior between particles, while the Bonding V2 model was used to characterize the agglomeration of the seedling pot, as well as the continuous structure and local failure behavior of the leaf, petiole and stem [33,34,35].

## 4. Parameter Calibration Test of Discrete Element Model

Because the structural morphology and loading characteristics of different components of Shanghai bok choy plug seedling differ markedly, and some microscopic parameters are difficult to measure directly, parameter calibration is required to improve the ability of the discrete element model to characterize its actual mechanical response and structural behavior. This study focused on calibrating the normal stiffness per unit area, tangential stiffness per unit area, critical normal stress, critical tangential stress and contact radius in the Bonding V2 model. These parameters directly affect the bonding strength, deformation capacity and failure characteristics between particles and, therefore, need to be inversely determined using a test–simulation comparison method. Contact parameters, such as the restitution coefficient, static friction coefficient and dynamic friction coefficient, were obtained from physical tests and could be directly used as input parameters for the Hertz–Mindlin (no slip) model.

### 4.1. Calibration Test of Seedling Pot Discrete Element Model

**Figure 38:**
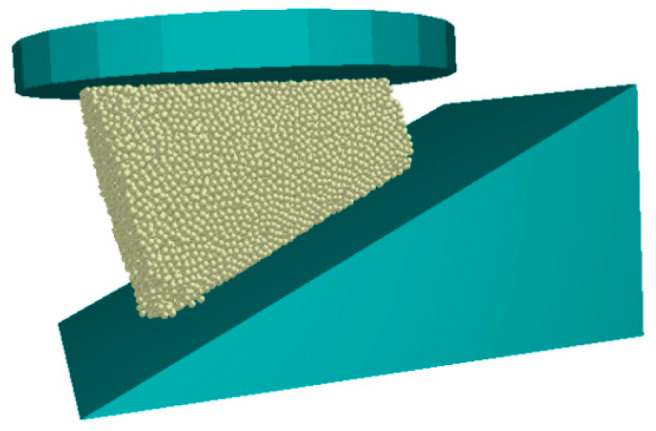
Schematic diagram of seedling pot flat plate compression simulation test.

To calibrate the mechanical parameters of the Bonding V2 model for the seedling pot discrete element model, a seedling pot flat plate compression simulation test was conducted. To ensure the reliability of the calibration results, the simulation conditions were kept consistent with those of the physical test. A three-dimensional model of the test device was established in SolidWorks 2016, saved in STEP format, and imported into EDEM 2024. The inclination angle of the cushion block was 11°, the diameter of the circular flat plate probe was 75 mm, the loading speed was 1 mm/s, and the compression displacement was 10 mm. The load variation in the probe during loading was monitored in real time. The test is shown in Figure 38.

#### 4.1.1. Screening and Analysis of Significant Factors

The ultimate compressive failure load can characterize the overall load-bearing capacity of the seedling pot and is relatively sensitive to changes in the mechanical parameters of the Bonding V2 model. Therefore, it was used as the response index for seedling pot parameter calibration. The normal stiffness per unit area *x*_1_, tangential stiffness per unit area *x*_2_, critical normal stress *x*_3_, critical tangential stress *x*_4_, and contact radius *x*_5_ were selected as test factors, and a Plackett–Burman test was conducted to screen significant factors [36,37]. The experimentally measured ultimate compressive failure load *F*_1_ of the seedling pot was 14.41 N. *x*_1_ was calculated according to Formula (15), and *x*_2_ was calculated according to Formulas (16) and (17). The remaining parameters were determined by referring to the relevant literature [38,39]. The Poisson’s ratio was 0.24, *x*_3_ was 0.03–0.10 MPa, *x*_4_ was 0.02–0.08 MPa, the particle radius was 0.5 mm, and *x*_5_ was 0.6–0.65 mm. After calculation, *x*_1_ was 2.84 × 10^9^–3.16 × 10^9^ N·m^−3^, and *x*_2_ was 1.15 × 10^9^–1.27 × 10^9^ N·m^−3^. The coded levels of the test factors are shown in Table 5.

(15) $$k_{\mathrm{nw}}=\frac{E_{w}}{L_{\mathrm{bw}}}$$

where *k*_nw_ is the normal stiffness per unit area of the material, N·m^−3^; *E*_w_ is the elastic modulus of the material, MPa; and *L*_bw_ is the center distance between adjacent discrete element particles, mm, taken as 1 mm.

(16) $$G_{w}=\frac{E_{w}}{2\left(1+\nu_{w}\right)}$$

(17) $$k_{\mathrm{tw}}=\frac{G_{w}}{L_{\mathrm{bw}}}$$

where *G*_w_ is the shear modulus of the material, MPa; *ν*_w_ is the Poisson’s ratio of the material; and *k*_tw_ is the tangential stiffness per unit area of the material, N·m^−3^.

According to Table 5, 12 groups of Plackett–Burman simulation tests were designed using Design-Expert 13 software. Each group was repeated 10 times, and the average value was taken as the final response value. The test scheme and results are shown in Table 6.

According to Table 7, the effect of x_1_ on the ultimate compressive failure load *F*_1_ of the seedling pot reached a highly significant level (*p* < 0.0001); the effects of *x*_3_ and *x*_4_ on *F*_1_ reached significant levels (*p* < 0.05), while the effects of *x*_2_ and *x*_5_ on *F*_1_ were not significant (*p* > 0.05). The contribution rates of the factors followed the order of *x*_1_ (76.39%), *x*_3_ (12.58%) and *x*_4_ (8.86%). Therefore, *x*_1_, *x*_3_ and *x*_4_ were selected as the main factors for the subsequent steepest ascent test and Box–Behnken response surface test. The seedling pot is a porous composite composed of roots and substrate particles. During compression, it mainly exhibits particle contact, pore compression, particle rearrangement and interlocking constraints between the roots and substrate. The normal stiffness per unit area directly affects the compressive deformation resistance of the particle system, while the critical normal stress and critical tangential stress reflect the failure thresholds of bonds under normal tensile–compressive and tangential shear actions, respectively. Therefore, these parameters had relatively significant effects on the ultimate compressive failure load of the seedling pot.

#### 4.1.2. Determination and Analysis of the Optimal Ranges of Significant Factors

Based on the screening results of the Plackett–Burman test, a steepest ascent test was conducted for *x*_1_, *x*_3_ and *x*_4_. The relative error between the simulation and physical test results of the seedling pot flat plate compression test was used as the evaluation index. Each group was repeated 10 times, and the average value was taken as the final response value. The test design and results are shown in Table 8. As *x*_1_, *x*_3_ and *x*_4_ increased, the ultimate compressive failure load gradually increased, while the relative error first decreased and then increased. Therefore, the 4th group was selected as the center level, and the 3rd and 5th groups were selected as the low and high levels, respectively, for the Box–Behnken response surface test [40,41,42].

#### 4.1.3. Establishment and Analysis of Regression Model

Using *x*_1_, *x*_3_ and *x*_4_ as test factors and *F*_1_ as the response value, a three-factor and three-level Box–Behnken response surface test was conducted. The coded levels of the factors are shown in Table 9.

According to Table 9, the Box–Behnken response surface test design for seedling pot flat plate compression was conducted using Design-Expert 13 software. Each group was repeated 10 times, and the average value was taken as the response value. The test scheme and results are shown in Table 10. Regression fitting analysis was performed on the test results, and a quadratic polynomial regression model of the ultimate compressive failure load *F*_1_ of the seedling pot was established, as shown in Formula (18).

(18) $$\begin{array}{c} F_{1}=14.68+1.043x_{1}+0.22x_{2}+0.1775x_{4}+0.29x_{1}x_{3}+0.355x_{1}x_{4}\\ +0.19x_{3}x_{4}-0.6725x_{1}^{2}+0.0625x_{3}^{2}-0.0025x_{4}^{2} \end{array}$$

Analysis of variance was performed on the test results, as shown in Table 11. The model had *p* < 0.0001, the *p* value of the lack-of-fit term was greater than 0.05, the lack-of-fit term was not significant, *R*^2^ = 0.9762, and CV = 1.435%, indicating that the regression model had good fitting performance and high reliability. The effects of *x*_1_ and the quadratic term *x*_1_^2^ on *F*_1_ were highly significant (*p* < 0.01); the effects of *x*_3_, *x*_4_ and the interaction terms *x*_1_*x*_3_ and *x*_1_*x*_4_ were significant (0.01 < *p* < 0.05); and the effects of the other terms were not significant. The order of single-factor effects was *x*_1_ > *x*_3_ > *x*_4_, and the order of interaction effects was *x*_1_*x*_4_ > *x*_1_*x*_3_.

#### 4.1.4. Optimization and Validation of Discrete Element Parameters for the Ultimate Compressive Failure Load of Seedling Pot

Using the measured ultimate compressive failure load of the seedling pot, 14.41 N, as the target, optimization was performed using the Optimization-Numerical module in Design-Expert. The optimized normal stiffness per unit area was 3.030 × 10^9^ N·m^−3^, the critical normal stress was 0.071 MPa, and the critical tangential stress was 0.054 MPa. The other non-significant parameters were set to intermediate values, with the tangential stiffness per unit area of 1.210 × 10^9^ N·m^−3^ and the contact radius of 0.625 mm. The optimized parameters were used to conduct the seedling pot flat plate compression simulation test, which was repeated 10 times. The mean ultimate compressive failure load of the seedling pot was 14.24 N, with a standard deviation of 0.4642 N and a coefficient of variation of 3.26%. The simulation results showed low dispersion and good stability and repeatability. The relative error compared with the experimental value was 1.19%, indicating that the established regression model for the ultimate compressive failure load of the seedling pot had good fitting performance. The discrete element parameters of the seedling pot are shown in Table 12.

### 4.2. Calibration Test of Leaf Discrete Element Model

**Figure 39:**
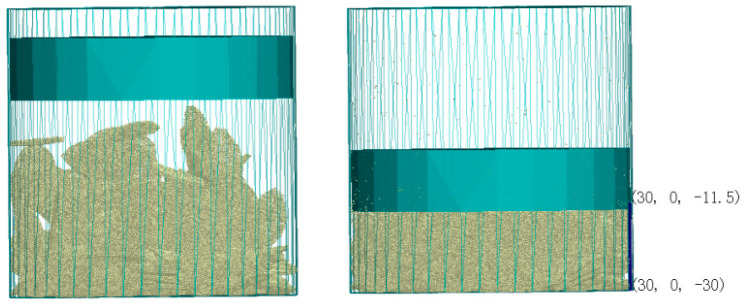
Schematic diagram of leaf cylinder compression simulation test.

To calibrate the mechanical parameters of the Bonding V2 model for the leaf discrete element model, a leaf cylinder compression simulation test was conducted. To ensure the reliability of the calibration results, the simulation conditions were kept consistent with those of the physical test. A three-dimensional model of the cylinder compression device was established in SolidWorks 2016 and imported into EDEM 2024. The stainless-steel cylinder had a radius of 25 mm and a height of 100 mm, and the cylindrical compression block had a radius of 24.5 mm and a height of 20 mm. Since the differences in elastic modulus and compression density between the large and small leaves were small, their mean values were used to uniformly characterize the leaf parameters. A particle factory was set inside the cylinder to generate a leaf discrete element model with a total mass of 10 g, including 5 g of large leaves and 5 g of small leaves. The compression block moved downward uniformly along the negative *Y*-axis direction at a speed of 0.01 m/s. When the load on the compression block reached 4.9 N, namely the gravity generated by the 500 g compression block, the distance between the bottom surface of the compression block and the bottom surface of the cylinder was recorded. The simulation test is shown in Figure 39.

#### 4.2.1. Screening and Analysis of Significant Factors

Leaf compression density can directly reflect the mechanical properties of leaf particles during compression and is highly sensitive to changes in the mechanical parameters of the Bonding V2 model. Therefore, it was used as the response index for leaf parameter calibration, and the compression density *ρ*_y_ was calculated according to Formula (4). The normal stiffness per unit area *x*_6_, tangential stiffness per unit area *x*_7_, critical normal stress *x*_8_, critical tangential stress *x*_9_, and contact radius *x*_10_ were selected as test factors, and a Plackett–Burman test was conducted to screen significant factors. The experimentally measured leaf compression density *ρ*_y_ was 0.268 g·cm^−3^. *x*_6_ was calculated according to Formula (15), and *x*_7_ was calculated according to Formulas (16) and (17). The remaining parameters were determined by referring to the relevant literature [31,43]. The Poisson’s ratio of the leaf was 0.32, *x*_8_ was 2–10 MPa, *x*_9_ was 1–6 MPa, the particle radius was 0.2 mm, and *x*_10_ was 0.24–0.26 mm. After calculation, *x*_6_ was 4.94 × 10^9^–5.55 × 10^9^ N·m^−3^, and *x*_7_ was 1.87 × 10^9^–2.10 × 10^9^ N·m^−3^. The coded levels of the test factors are shown in Table 13.

According to Table 13, 12 groups of Plackett–Burman simulation tests were designed using Design-Expert 13. Each group was repeated 10 times, and the average value was taken as the response value. The test scheme and results are shown in Table 14.

According to Table 15, the effects of *x*_6_ and *x*_8_ on the leaf compression density *ρ*_y_ reached a highly significant level (*p* < 0.01); the effect of *x*_10_ on *ρ*_y_ reached a significant level (*p* < 0.05); while the effects of *x*_7_ and *x*_9_ on *ρ*_y_ were not significant (*p* > 0.05). Further comparison showed that the contribution rates of the factors followed the order of *x*_6_ (69.35%), *x*_8_ (24.97%) and *x*_10_ (2.77%). Therefore, *x*_6_, *x*_8_ and *x*_10_ were selected as the main factors for the subsequent steepest ascent test and Box–Behnken response surface test. The leaf is a thin, flexible structure that mainly undergoes bending, stacking and local contact compression during cylinder compression. The normal stiffness per unit area affects the compressive deformation resistance between leaf particles, the critical normal stress determines the failure threshold of bonds under compression, and the contact radius affects the effective bonding range between particles and stacking stability. Therefore, *x*_6_, *x*_8_ and *x*_10_ had obvious effects on leaf compression density.

#### 4.2.2. Determination and Analysis of the Optimal Ranges of Significant Factors

A steepest ascent test was conducted for *x*_6_, *x*_8_ and *x*_10_. The relative error between the leaf cylinder compression simulation test results and the physical test results was used as the evaluation index. Each group was repeated 10 times, and the average value was taken as the final response value. The test design and results are shown in Table 16. As *x*_6_, *x*_8_ and *x*_10_ increased, *ρ*_y_ also gradually increased, while the relative error first decreased and then increased. Therefore, the 3rd group was selected as the center level, and the 2nd and 4th groups were selected as the low and high levels, respectively, for the Box–Behnken response surface test.

#### 4.2.3. Establishment and Analysis of Regression Model

Using *x*_6_, *x*_8_ and *x*_10_ as test factors and *ρ*_y_ as the response value, a three-factor and three-level Box–Behnken response surface test was conducted. The coded levels of the test factors are shown in Table 17.

According to Table 17, the Box–Behnken response surface test design for leaf cylinder compression was conducted using Design-Expert 13 software. Each group was repeated 10 times, and the average value was taken as the response value. The test scheme and results are shown in Table 18. Regression fitting analysis was performed on the test results, and a quadratic polynomial regression model of leaf compression density *ρ*_y_ was established, as shown in Formula (19).

(19) $$\begin{array}{c} \rho_{y}=0.2791+0.01724x_{6}+0.004862x_{8}+0.004350x_{10}\\ +0.006350x_{6}x_{8}+0.005425x_{6}x_{10}+0.0001250x_{8}x_{10}\\ -0.01850x_{6}^{2}-0.006945x_{8}^{2}-0.001070x_{10}^{2} \end{array}$$

Analysis of variance was performed on the test data, as shown in Table 19. The results showed that the model had a *p* value < 0.0001, indicating that the model was significant. The *p* value of the lack-of-fit term was greater than 0.05, indicating that the lack-of-fit term was not significant. The coefficient of determination *R*^2^ was 0.9813, and the coefficient of variation CV was 1.347%, indicating that the regression model had good fitting performance and high reliability. The effects of *x*_6_ and *x*_6_^2^ on *ρ*_y_ reached a highly significant level (*p* < 0.0001). The effects of *x*_8_, *x*_8_^2^ and *x*_6_*x*_8_ on *ρ*_y_ reached a highly significant level (*p* < 0.001). The effects of *x*_10_ and *x*_6_*x*_10_ on *ρ*_y_ reached a significant level (0.01 < *p* < 0.05), while the effects of *x*_8_*x*_10_ and *x*_10_^2^ on ρ<sub>y</sub> were not significant (*p* > 0.05). The order of significant effects of single factors was *x*_6_ > *x*_8_ > *x*_10_; the order of significant effects of interaction terms was *x*_6_*x*_8_ > *x*_6_*x*_10_; and the order of significant effects of quadratic terms was *x*_6_^2^ > *x*_8_^2^.

#### 4.2.4. Optimization and Validation of Discrete Element Parameters for Leaf Compression Density

Using the experimentally measured leaf compression density of 0.268 g·cm^−3^ as the target, optimization was performed using the Optimization-Numerical module in Design-Expert 13 software. The optimized normal stiffness per unit area was 5.23 × 10^9^ N·m^−3^, the critical normal stress was 4.189 MPa, and the contact radius was 0.251 mm. The other non-significant parameters were set to intermediate values, with the tangential stiffness per unit area of 1.99 × 10^9^ N·m^−3^ and the critical tangential stress of 3.5 MPa. The optimized parameters were used to conduct the leaf cylinder compression simulation test, which was repeated 10 times. The mean leaf compression density was 0.265 g·cm^−3^, with a standard deviation of 0.0109 g·cm^−3^ and a coefficient of variation of 3.26%. The simulation results showed low dispersion and good stability and repeatability. The relative error was 1.13%, indicating that the established regression model for leaf compression density had good fitting performance. The discrete element parameters of the leaf are shown in Table 20.

### 4.3. Calibration Test of Petiole Discrete Element Model

**Figure 40:**
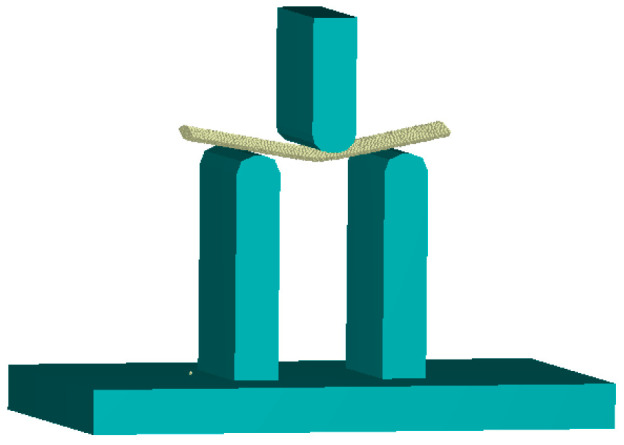
Schematic diagram of petiole three-point bending simulation test.

To calibrate the mechanical parameters of the Bonding V2 model for the petiole discrete element model, a petiole three-point bending simulation test was conducted. The simulation conditions were kept consistent with those of the physical test. Taking the large petiole as an example, a three-dimensional model of the petiole three-point bending test device was established in SolidWorks 2016 software and imported into EDEM 2024. The support span was 10 mm, and the 25 mm long petiole discrete element model was placed on the two supports. The loading probe moved vertically downward at a speed of 1 mm/s to apply a bending load to the middle of the petiole. The loading displacement was set to 4 mm, and the load variation in the probe during loading was monitored in real time. The simulation test is shown in Figure 40.

#### 4.3.1. Screening and Analysis of Significant Factors

The ultimate bending failure load of the petiole can characterize its ultimate load-bearing capacity and damage failure characteristics, and is relatively sensitive to changes in the Bonding V2 parameters. Therefore, it was used as the response index for petiole parameter calibration. The normal stiffness per unit area *x*_11_, tangential stiffness per unit area *x*_12_, critical normal stress *x*_13_, critical tangential stress *x*_14_, and contact radius *x*_15_ were selected as test factors, and a Plackett–Burman test was conducted to screen significant factors. The experimentally measured ultimate bending failure load *F*_2_ of the petiole was 3.07 N. *x*_11_ was calculated according to Formula (15), and *x*_12_ was calculated according to Formulas (16) and (17). The remaining parameters were determined by referring to relevant literature [31,43]. The Poisson’s ratio of the petiole was 0.33, *x*_13_ was 8−18 MPa, *x*_14_ was 4–12 MPa, the particle radius was 0.2 mm, and *x*_15_ was 0.24–0.26 mm. After calculation, *x*_11_ was 4.095 × 10^10^–4.153 × 10^10^ N·m^−3^, and *x*_12_ was 1.540 × 10^10^–1.561 × 10^10^ N·m^−3^. The coded levels of the test factors are shown in Table 21.

According to Table 21, 12 groups of Plackett–Burman simulation tests were designed using Design-Expert 13. Each group was repeated 10 times, and the average value was taken as the response value. The test scheme and results are shown in Table 22.

According to Table 23, the effects of *x*_12_ and *x*_15_ on the ultimate bending failure load *F*_2_ of the petiole reached a highly significant level (*p* < 0.001); the effect of *x*_11_ on *F*_2_ reached a significant level (0.01 < *p* < 0.05); while the effects of *x*_13_ and *x*_14_ on *F*_2_ were not significant (*p* > 0.05). The contribution rates of the factors followed the order of *x*_12_ (60.64%), *x*_15_ (29.98%) and *x*_11_ (4.53%). Therefore, *x*_12_, *x*_15_ and *x*_11_ were selected as the main factors for the subsequent steepest ascent test and Box–Behnken response surface test. The petiole is a slender, flexible organ with an approximately elliptical cross-section. During bending loading, relative dislocation and shear deformation are likely to occur between the particle layers on the tensile and compressive sides. The tangential stiffness per unit area directly affects the ability of particles to resist tangential sliding and bending deformation, and therefore has the greatest effect on the ultimate bending failure load of the petiole. The contact radius affects the effective action range of bonds between adjacent particles, while the normal stiffness per unit area affects the overall deformation resistance of the petiole during bending. Therefore, both parameters also showed significant effects.

#### 4.3.2. Determination and Analysis of the Optimal Ranges of Significant Factors

A steepest ascent test was conducted for *x*_11_, *x*_12_ and *x*_15_. The relative error between the petiole three-point bending simulation test results and the physical test results was used as the evaluation index. Each group was repeated 10 times, and the average value was taken as the final response value. The design and results of the steepest ascent simulation test are shown in Table 24. As *x*_11_, *x*_12_ and *x*_15_ increased, *F*_2_ also gradually increased, while the relative error first decreased and then increased. Therefore, the 3rd group was selected as the center level, and the 2nd and 4th groups were selected as the low and high levels, respectively, for the Box–Behnken response surface test.

#### 4.3.3. Establishment and Analysis of Regression Model

Using *x*_11_, *x*_12_ and *x*_15_ as test factors and *F*_2_ as the response value, a three-factor and three-level Box–Behnken response surface test was conducted. The coded levels of the test factors are shown in Table 25.

According to Table 25, the Box–Behnken response surface test design for petiole three-point bending was conducted using Design-Expert 13 software. Each group was repeated 10 times, and the average value was taken as the response value. The test scheme and results are shown in Table 26. Regression fitting analysis was performed on the test results, and a quadratic polynomial regression model of the ultimate bending failure load *F*_2_ of the petiole was established, as shown in Formula (20).

(20) $$\begin{array}{c} F_{2}=3.070+0.5263x_{11}+0.3144x_{12}+0.2760x_{15}\\ -0.0002500x_{11}x_{12}-0.03900x_{11}x_{15}+0.08600x_{12}x_{15}\\ -0.02138x_{11}^{2}+0.05913x_{12}^{2}-0.1626x_{15}^{2} \end{array}$$

Analysis of variance was performed on the test data, as shown in Table 27. The results showed that the model had a *p* value < 0.0001, indicating that the model was significant. The *p* value of the lack-of-fit term was greater than 0.05, indicating that the lack-of-fit term was not significant. The coefficient of determination R^2^ was 0.9902, and CV was 1.567%, indicating that the regression model had good fitting performance and high reliability. The effects of x_12_ and x_15_ on F_2_ reached a highly significant level (*p* < 0.0001). The effect of x_15_^2^ on F_2_ reached a highly significant level (*p* < 0.001). The effects of x_11_, x_12_x_15_ and x_12_^2^ on F_2_ reached a significant level (0.01 < *p* < 0.05), while the effects of x_11_x_12_, x_11_x_15_ and x_11_^2^ on F_2_ were not significant (*p* > 0.05). The order of significant effects of single factors was x_12_ > x_15_ > x_11_, and the order of significant effects of quadratic terms was x_15_^2^ > x_12_^2^.

#### 4.3.4. Optimization and Validation of Discrete Element Parameters for the Ultimate Bending Failure Load of Petiole

Using the experimentally measured ultimate bending failure load of the petiole, 3.07 N, as the target, optimization was performed using the Optimization-Numerical module in Design-Expert software. The optimized normal stiffness per unit area was 4.112 × 10^10^ N·m^−3^, the tangential stiffness per unit area was 1.552 × 10^10^ N·m^−3^, and the contact radius was 0.249 mm. The other non-significant parameters were set to intermediate values, with the critical normal stress of 13 MPa and the critical tangential stress of 8 MPa. The optimized parameters were used to conduct the petiole three-point bending simulation test, which was repeated 10 times. The mean ultimate bending failure load of the petiole was 3.04 N, with a standard deviation of 0.0879 N and a coefficient of variation of 2.89%. The simulation results showed low dispersion and good stability and repeatability. The relative error compared with the experimental value was 0.99%, indicating that the established regression model for the ultimate bending failure load of the petiole had good fitting performance. The parameter calibration method for the small petiole was consistent with that for the large petiole and is not repeated here. The discrete element parameters of the petiole are shown in Table 28.

### 4.4. Calibration Test of Stem Discrete Element Model

**Figure 41:**
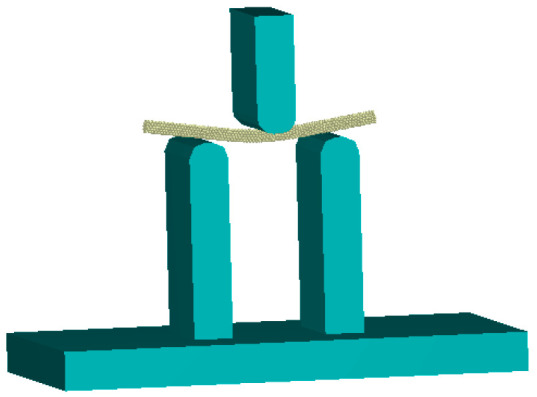
Schematic diagram of stem three-point bending simulation test.

To calibrate the mechanical parameters of the Bonding V2 model for the stem discrete element model, a stem three-point bending simulation test was conducted. To ensure the reliability of the calibration results, the simulation conditions were kept consistent with those of the physical test. The test device was consistent with that used in the petiole three-point bending simulation. A 20 mm long stem discrete element model was placed on the two supports, with the loading direction perpendicular to the stem axis. The loading probe moved vertically downward at a speed of 1 mm/s to apply a bending load to the middle of the stem. The loading displacement was set to 5 mm. When the load detected by the probe reached 0.1 N, data acquisition began, and the load variation in the probe during loading was monitored in real time. The simulation test is shown in Figure 41.

#### 4.4.1. Screening and Analysis of Significant Factors

The ultimate bending failure load of the stem can characterize its ultimate load-bearing capacity and damage failure characteristics, and is relatively sensitive to changes in the Bonding V2 parameters. Therefore, it was used as the response index for stem parameter calibration. The normal stiffness per unit area *x*_16_, tangential stiffness per unit area *x*_17_, critical normal stress *x*_18_, critical tangential stress *x*_19_, and contact radius *x*_20_ were selected as test factors, and a Plackett–Burman test was conducted to screen significant factors. The experimentally measured ultimate bending failure load *F*_3_ of the stem was 4.15 N. *x*_16_ was calculated according to Formula (15), and *x*_17_ was calculated according to Formulas (16) and (17). The remaining parameters were determined by referring to the relevant literature [31,43]. The Poisson’s ratio of the stem was 0.38, *x*_18_ was 15–25 MPa, *x*_19_ was 10–20 MPa, the particle radius was 0.2 mm, and *x*_20_ was 0.24–0.26 mm. After calculation, *x*_16_ was 8.193 × 10^10^–8.403 × 10^10^ N·m^−3^, and *x*_17_ was 2.968 × 10^10^–3.044 × 10^10^ N·m^−3^. The coded levels of the test factors are shown in Table 29.

According to Table 29, 12 groups of Plackett–Burman simulation tests were designed using Design-Expert 13. Each group was repeated 10 times, and the average value was taken as the response value. The test scheme and results are shown in Table 30.

According to Table 31, the effects of *x*_18_ and *x*_20_ on the ultimate bending failure load *F*_3_ of the stem reached a highly significant level (*p* < 0.0001); the effect of *x*_16_ on *F*_3_ reached a highly significant level (*p* < 0.001), while the effects of *x*_17_ and *x*_19_ on *F*_3_ were not significant (*p* > 0.05). The contribution rates of the factors followed the order of *x*_18_ (63.17%), *x*_20_ (33.20%) and *x*_16_ (2.37%). Therefore, *x*_16_, *x*_18_ and *x*_20_ were selected as the main factors for the subsequent steepest ascent test and Box–Behnken response surface test. The stem is the main load-bearing structure connecting the seedling pot and the leaf part, with a denser tissue structure and higher vascular bundle content than the petiole. During bending failure, tissue cracking on the tensile side of the stem and normal fracture of bonds are more prominent. Therefore, the critical normal stress became the main factor affecting the ultimate bending failure load of the stem. The contact radius affects the bonding range and load transfer capacity between particles, while the normal stiffness per unit area affects the overall bending deformation resistance of the stem. Therefore, both parameters also had significant effects on the bending failure response of the stem.

#### 4.4.2. Determination and Analysis of the Optimal Ranges of Significant Factors

To determine the optimal ranges of the factors, a steepest ascent test was conducted for *x*_16_, *x*_18_ and *x*_20_. The relative error between the stem three-point bending simulation test results and the physical test results was used as the evaluation index. Each group was repeated 10 times, and the average value was taken as the final response value. The test design and results are shown in Table 32. As *x*_16_, *x*_18_ and *x*_20_ increased, the ultimate bending failure load *F*_3_ of the stem also gradually increased, while the relative error first decreased and then increased. Therefore, the 3rd group was selected as the center level, and the 2nd and 4th groups were selected as the low and high levels, respectively, for the Box–Behnken response surface test.

#### 4.4.3. Establishment and Analysis of Regression Model

Using *x*_16_, *x*_18_ and *x*_20_ as test factors and *F*_3_ as the response value, a three-factor and three-level Box–Behnken response surface test was conducted. The coded levels of the test factors are shown in Table 33.

According to Table 33, the Box–Behnken response surface test design for stem three-point bending was conducted using Design-Expert 13 software. Each group was repeated 10 times, and the average value was taken as the response value. The test scheme and results are shown in Table 34. Regression fitting analysis was performed on the test results, and a quadratic polynomial regression model of the ultimate bending failure load *F*_3_ of the stem was established, as shown in Formula (21).

(21) $$\begin{array}{c} F_{3}=4.1680+0.04875x_{16}+0.2625x_{18}+0.15625x_{20}\\ +0.0225x_{16}x_{18}+0.0650x_{16}x_{20}-0.0075x_{18}x_{20}\\ +0.0560x_{16}^{2}-0.2065x_{18}^{2}-0.0640x_{20}^{2} \end{array}$$

Analysis of variance was performed on the test data, as shown in Table 35. The results showed that the model had a *p* value < 0.0001, indicating that the model was significant. The *p* value of the lack-of-fit term was greater than 0.05, indicating that the lack-of-fit term was not significant. The coefficient of determination *R*^2^ was 0.9825, and CV was 1.237%, indicating that the regression model had good fitting performance and high reliability. The effects of *x*_18_, *x*_20_ and *x*_18_^2^ on *F*_3_ reached a highly significant level (*p* < 0.0001). The effects of *x*_16_, *x*_16_*x*_20_ and *x*_20_^2^ on *F*_3_ reached a significant level (0.01 < *p* < 0.05), while the effects of *x*_16_*x*_18_, *x*_18_*x*_20_ and *x*_16_^2^ on *F*_3_ were not significant (*p* > 0.05). The order of significant effects of single factors was *x*_18_ > *x*_20_ > *x*_16_, and the order of significant effects of quadratic terms was *x*_18_^2^ > *x*_20_^2^.

#### 4.4.4. Optimization and Validation of Discrete Element Parameters for the Ultimate Bending Failure Load of Stem

Using the experimentally measured ultimate bending failure load of the stem, 4.15 N, as the target, optimization was performed using the Optimization-Numerical module in Design-Expert 13 software. The optimized normal stiffness per unit area was 8.298 × 10^10^ N·m^−3^, the critical normal stress was 22.42 MPa, and the contact radius was 0.248 mm. The other non-significant parameters were set to intermediate values, with the tangential stiffness per unit area of 3.006 × 10^10^ N·m^−3^ and the critical tangential stress of 15 MPa. The optimized parameters were used to conduct the stem three-point bending simulation test, which was repeated 10 times. The mean ultimate bending failure load of the stem was 4.12 N, with a standard deviation of 0.1442 N and a coefficient of variation of 3.50%. The simulation results showed low dispersion and good stability and repeatability. The relative error compared with the experimental value was 0.72%, indicating that the established regression model for the ultimate bending failure load of the stem had good fitting performance. The discrete element parameters of the stem are shown in Table 36.

### 4.5. Discussion

Shanghai bok choy plug seedling is composed of multiple components, including the seedling pot, leaf, petiole and stem, which differ markedly in structural morphology and mechanical response. If a single homogeneous model is used, it is difficult to accurately characterize the stress and deformation characteristics of the whole plug seedling during automatic transplanting. Therefore, in this study, a combined method of component-wise modeling and overall reconstruction was adopted, and the Hertz–Mindlin (no slip) and Bonding V2 models were used in combination. This approach can describe the contact behavior between particles and characterize the continuous structure and failure process of each component, thereby improving the authenticity of the whole-plant discrete element model of Shanghai bok choy plug seedling.

The significant parameters differed among different components, indicating that their dominant mechanical mechanisms were not the same. The compression response of the seedling pot was mainly affected by the stiffness and bonding strength of the particle system. The compression and stacking characteristics of the leaf were closely related to the normal stiffness, bonding strength and contact range. Under bending loads, the petiole and stem were affected by bonding stiffness, bonding strength and contact radius, respectively. Therefore, for multi-component composite biological materials such as Shanghai bok choy plug seedling, overall calibration using unified parameters is difficult to fully reflect the actual mechanical properties of each organ. It is more reasonable to conduct component-wise parameter calibration according to the structural characteristics and typical loading forms of different components.

After validation using the optimized parameters, the relative errors between the simulation results and physical test results of the seedling pot, leaf, petiole and stem were all less than 1.20%, indicating that the established model had good accuracy and could provide a basis for subsequent interaction simulation between Shanghai bok choy plug seedling and key components of automatic transplanting equipment. It should be noted that the parameters in this study were mainly obtained under specific variety, seedling cultivation period and moisture content conditions. Further validation and correction of the dynamic response of the whole-plant model are still needed in combination with different seedling cultivation conditions and actual transplanting processes.

## 5. Conclusions

To address the significant differences in the structural and mechanical properties of different components of Shanghai bok choy plug seedling, as well as the lack of an accurate and reliable whole-plant discrete element model and key bonding parameters for automatic transplanting process simulation, a 128-cell Shanghai bok choy plug seedling was selected as the research object. Material property determination, whole-plant discrete element model construction, key parameter calibration and simulation validation were carried out. The main conclusions are as follows:

(1) The morphological, physical, mechanical and contact properties of Shanghai bok choy plug seedling were systematically determined, and basic parameters such as geometric dimensions, density, elastic modulus, static friction coefficient, dynamic friction coefficient and collision restitution coefficient of each component were obtained. The results showed that the different components of Shanghai bok choy plug seedling differed markedly in structural morphology, material properties and mechanical response. The ultimate compressive failure load of the seedling pot was 14.41 N, the leaf compression density was 0.268 g·cm^−3^, the ultimate bending failure loads of the large and small petioles were 3.07 N and 1.76 N, respectively, and the ultimate bending failure load of the stem was 4.15 N, indicating that it is necessary to characterize the heterogeneity of the whole plug seedling using a component-wise modeling method.

(2) Considering the multi-component composition and strong structural continuity of Shanghai bok choy plug seedling, a whole-plant discrete element modeling method combining component-wise modeling and overall reconstruction was proposed. By separately obtaining the spatial particle coordinates of the seedling pot, leaf, petiole and stem and splicing them in a unified coordinate system, a whole-plant discrete element model of Shanghai bok choy plug seedling was established. The combined Hertz–Mindlin (no slip) and Bonding V2 contact models were adopted to simultaneously characterize interparticle contact, tissue continuity and local failure behavior.

(3) Based on the Plackett–Burman test, steepest ascent test and Box–Behnken response surface test, the screening and optimized calibration of key bonding parameters for each component were completed. The results showed that the significant parameters affecting the ultimate compressive failure load of the seedling pot were normal stiffness per unit area, critical normal stress and critical tangential stress, with optimized values of 3.030 × 10^9^ N·m^−3^, 0.071 MPa and 0.054 MPa, respectively. The significant parameters affecting leaf compression density were normal stiffness per unit area, critical normal stress and contact radius, with optimized values of 5.23 × 10^9^ N·m^−3^, 4.189 MPa and 0.251 mm, respectively. The significant parameters affecting the ultimate bending failure load of the petiole were tangential stiffness per unit area, contact radius and normal stiffness per unit area, with optimized values of 1.552 × 10^10^ N·m^−3^, 0.249 mm and 4.112 × 10^10^ N·m^−3^, respectively. The significant parameters affecting the ultimate bending failure load of the stem were critical normal stress, contact radius and normal stiffness per unit area, with optimized values of 22.42 MPa, 0.248 mm and 8.298 × 10^10^ N·m^−3^, respectively.

(4) Simulation validation was conducted using the optimized parameters. The relative errors between the simulated and physical test values of the ultimate compressive failure load of the seedling pot, leaf compression density, ultimate bending failure load of the petiole, and ultimate bending failure load of the stem were 1.19%, 1.13%, 0.99% and 0.72%, respectively. The results indicated that the established whole-plant discrete element model of Shanghai bok choy plug seedling could accurately characterize the mechanical response characteristics of each component. This model can provide a basis for subsequent discrete element simulation of the interaction between Shanghai bok choy plug seedling and key components of automatic transplanting equipment, as well as for the design optimization of automatic transplanting equipment.

## Figures and Tables

**Table 1:** Determination results of morphological parameters of Shanghai bok choy plug seedling.

Parameter	Mean Value	Standard Deviation	Coefficient of Variation
Total height of plug seedling *H*_z_ (mm)	117.62	7.85	6.67%
Leaf spread of plug seedling *L*_z_ (mm)	124.74	6.97	5.59%
Total mass of plug seedling *m*_z_ (g)	11.873	0.86	7.24%
Height of seedling pot *H*_b_ (mm)	40.63	0.94	2.31%
Upper end-face length of seedling pot *L*_a1_ (mm)	31.37	0.41	1.31%
Lower end-face length of seedling pot *L*_a2_ (mm)	12.52	0.37	2.96%
Stem length *L*_j_ (mm)	22.46	1.61	7.17%
Stem diameter *d*_j_ (mm)	2.44	0.16	6.56%
Large leaf length *L*_x1_ (mm)	39.41	1.82	4.62%
Large petiole length *L*_x2_ (mm)	32.33	1.28	3.96%
Large leaf width *B*_x1_ (mm)	23.04	0.79	3.43%
Large petiole width *B*_x2_ (mm)	6.41	0.47	7.33%
Large leaf thickness *t*_x1_ (mm)	0.68	0.03	4.41%
Large petiole thickness *t*_x2_ (mm)	1.57	0.06	3.82%
Small leaf length *L*_y1_ (mm)	29.73	1.12	3.77%
Small petiole length *L*_y2_ (mm)	23.58	1.31	5.56%
Small leaf width *B*_y1_ (mm)	19.26	0.89	4.62%
Small petiole width *B*_y2_ (mm)	5.42	0.27	4.98%
Small leaf thickness t_y1_ (mm)	0.62	0.03	4.84%
Small petiole thickness *t*_y2_ (mm)	1.44	0.08	5.56%

**Table 2:** Determination results of static friction coefficient.

Object	Maximum Value	Minimum Value	Mean Value	Standard Deviation	Coefficient of Variation (%)
Seedling pot-Seedling pot	0.67	0.59	0.63	0.0238	3.78
Seedling pot-Steel plate	0.43	0.38	0.41	0.0140	3.42
Seedling pot-Plastic plate	0.48	0.42	0.45	0.0185	4.12
Leaf-Leaf	0.59	0.53	0.56	0.0120	3.57
Leaf-Steel plate	0.54	0.49	0.52	0.0166	3.18
Petiole-Petiole	0.51	0.45	0.48	0.0203	4.22
Petiole-Steel plate	0.52	0.47	0.50	0.0144	2.87
Stem-Stem	0.48	0.43	0.46	0.0166	3.61
Stem-Steel plate	0.52	0.46	0.49	0.0188	3.83

**Table 3:** Determination results of dynamic friction coefficient.

Object	Maximum Value	Minimum Value	Mean Value	Standard Deviation	Coefficient of Variation (%)
Seedling pot-Seedling pot	0.39	0.35	0.37	0.01247	3.37
Seedling pot-Steel plate	0.26	0.23	0.25	0.0082	3.29
Seedling pot-Plastic plate	0.28	0.25	0.27	0.0100	3.71
Leaf-Leaf	0.36	0.32	0.34	0.0123	3.63
Leaf-Steel plate	0.33	0.29	0.31	0.0129	4.16
Petiole-Petiole	0.30	0.27	0.29	0.0010	3.44
Petiole-Steel plate	0.34	0.30	0.32	0.0122	3.82
Stem-Stem	0.24	0.21	0.23	0.0093	4.03
Stem-Steel plate	0.30	0.24	0.28	0.0111	3.97

**Table 4:** Determination results of collision restitution coefficient.

Object	Maximum Value	Minimum Value	Mean Value	Standard Deviation	Coefficient of Variation (%)
Seedling pot-Seedling pot	0.25	0.22	0.24	0.0010	4.16
Seedling pot-Steel plate	0.61	0.54	0.58	0.0225	3.87
Seedling pot-Plastic plate	0.44	0.39	0.42	0.0147	3.51
Leaf-Leaf	0.14	0.12	0.13	0.0055	4.24
Leaf-Steel plate	0.39	0.34	0.37	0.0145	3.92
Petiole-Petiole	0.31	0.27	0.29	0.0126	4.33
Petiole-Steel plate	0.64	0.58	0.61	0.0220	3.61
Stem-Stem	0.34	0.30	0.32	0.0122	3.82
Stem-Steel plate	0.72	0.66	0.69	0.0229	3.32

**Table 5:** Coded levels of factors in the Plackett–Burman test.

Factor	Code	
	−1	1
Normal stiffness per unit area *x*_1_ (N·m^−3^)	2.84 × 10^9^	3.16 × 10^9^
Tangential stiffness per unit area *x*_2_ (N·m^−3^)	1.15 × 10^9^	1.27 × 10^9^
Critical normal stress *x*_3_ (MPa)	0.03	0.10
Critical tangential stress *x*_4_ (MPa)	0.02	0.08
Contact radius *x*_5_ (mm)	0.6	0.65

**Table 6:** Design and results of the Plackett–Burman test.

Serial Number	*x*_1_ (N·m^−3^)	*x*_2_ (N·m^−3^)	*x*_3_ (MPa)	*x*_4_ (MPa)	*x*_5_ (mm)	*F*_1_ (N)
1	−1	1	−1	1	1	13.72
2	1	−1	1	1	−1	16.84
3	−1	−1	−1	−1	−1	12.36
4	1	−1	1	1	1	16.65
5	−1	−1	1	−1	1	13.72
6	−1	1	1	−1	1	13.10
7	1	−1	−1	−1	1	15.17
8	1	1	−1	−1	−1	14.86
9	1	1	1	−1	−1	16.01
10	1	1	−1	1	1	15.55
11	−1	1	1	1	−1	14.39
12	−1	−1	−1	1	−1	13.08

**Table 7:** Significance analysis of the Plackett–Burman test.

Source	Sum of Squares	Degree of Freedom	Mean Square	*F*	*p*	Contribution Rate (%)	Significance Ranking
Model	23.11	5	4.622	49.45	<0.0001		
*x*_1_	18.03	1	18.03	193.0	<0.0001	76.39	1
*x*_2_	0.003008	1	0.003008	0.03219	0.8635	0.01	5
*x*_3_	2.97	1	2.97	31.78	0.001334	12.58	2
*x*_4_	2.092	1	2.092	22.38	0.003221	8.86	3
*x*_5_	0.01141	1	0.01141	0.1221	0.7387	0.05	4
Residual	0.5607	6	0.09345				
Total	23.67	11					

**Table 8:** Design and results of the steepest ascent test.

Serial Number	*x*_1_ (N·m^−3^)	*x*_3_ (MPa)	*x*_4_ (MPa)	*F*_1_ (N)	Relative Error (%)
1	2.840 × 10^9^	0.030	0.020	13.21	10.01
2	2.904 × 10^9^	0.044	0.032	13.88	5.45
3	2.968 × 10^9^	0.058	0.044	14.42	1.77
4	3.032 × 10^9^	0.072	0.056	14.66	0.14
5	3.096 × 10^9^	0.086	0.068	15.07	2.66
6	3.160 × 10^9^	0.100	0.080	15.63	6.47

**Table 9:** Coded levels of factors in the Box–Behnken response surface test.

Factor	Code		
	−1	0	1
Normal stiffness per unit area *x*_1_ (N·m^−3^)	2.968 × 10^9^	3.032 × 10^9^	3.096 × 10^9^
Critical normal stress *x*_3_ (MPa)	0.058	0.072	0.086
Critical tangential stress *x*_4_ (MPa)	0.044	0.056	0.068

**Table 10:** Design and results of the Box–Behnken response surface test.

Serial Number	Factor			*F*_1_ (N)
	*x*_1_	*x*_3_	*x*_4_	
1	0	−1	−1	14.52
2	0	0	0	14.36
3	0	0	0	14.84
4	1	1	0	15.75
5	1	0	−1	14.44
6	0	0	0	14.74
7	−1	1	0	12.87
8	0	0	0	14.90
9	1	0	1	15.44
10	0	1	−1	14.54
11	−1	0	−1	13.28
12	0	−1	1	14.56
13	−1	−1	0	12.97
14	0	1	1	15.34
15	−1	0	1	12.86
16	0	0	0	14.56
17	1	−1	0	14.69

**Table 11:** Analysis of variance of the Box–Behnken response surface test.

Source	Sum of Squares	Degree of Freedom	Mean Square	*F*	*p*
Model	12.23	9	1.359	31.87	<0.0001
*x*_1_	8.694	1	8.694	203.9	<0.0001
*x*_3_	0.3872	1	0.3872	9.080	0.0195702
*x*_4_	0.2520	1	0.2520	5.911	0.0453408
*x*_1_ *x*_3_	0.3364	1	0.3364	7.889	0.0261949
*x*_1_ *x*_4_	0.5041	1	0.5041	11.82	0.0108632
*x*_3_ *x*_4_	0.1444	1	0.1444	3.386	0.108316
*x*_1_^2^	1.904	1	1.904	44.66	0.000281948
*x*_3_^2^	0.01645	1	0.01645	0.3857	0.554244
*x*_4_^2^	0.00002632	1	0.00002632	0.0006171	0.980874
Residual	0.2985	7	0.04264		
Lack-of-fit term	0.1041	3	0.03470	0.7140	0.592863
Pure error	0.1944	4	0.04860		
Total	12.53	16			

**Table 12:** Discrete element parameters of seedling pot.

Object	Parameter	Value
Seedling pot	Density (g·cm^−3^)	0.794
	Poisson’s ratio	0.24
	Elastic modulus (MPa)	3.03
Steel plate	Density (g·cm^−3^)	7.89
	Poisson’s ratio	0.27
	Elastic modulus (MPa)	2.0 × 10^5^
Plastic plate	Density (g·cm^−3^)	1.05
	Poisson’s ratio	0.35
	Elastic modulus (MPa)	3.38 × 10^3^
Seedling pot-Seedling pot	Static friction coefficient	0.63
	Dynamic friction coefficient	0.37
	Collision restitution coefficient	0.24
	Normal stiffness per unit area (N·m^−3^)	3.030 × 10^9^
	Tangential stiffness per unit area (N·m^−3^)	1.21 × 10^9^
	Critical normal stress (MPa)	0.071
	Critical tangential stress (MPa)	0.054
	Contact radius (mm)	0.625
Seedling pot-Steel plate	Static friction coefficient	0.41
	Dynamic friction coefficient	0.25
	Collision restitution coefficient	0.58
Seedling pot-Plastic plate	Static friction coefficient	0.45
	Dynamic friction coefficient	0.27
	Collision restitution coefficient	0.42

**Table 13:** Coded levels of factors in the Plackett–Burman test.

Factor	Code	
	−1	1
Normal stiffness per unit area *x*_6_ (N·m^−3^)	4.94 × 109	5.55 × 109
Tangential stiffness per unit area *x*_7_ (N·m^−3^)	1.87 × 109	2.10 × 109
Critical normal stress *x*_8_ (MPa)	2	10
Critical tangential stress *x*_9_ (MPa)	1	6
Contact radius *x*_10_ (mm)	0.24	0.26

**Table 14:** Design and results of the Plackett–Burman test.

Serial Number	*x*_6_ (N·m^−3^)	*x*_7_ (N·m^−3^)	*x*_8_ (MPa)	*x*_9_ (MPa)	*x*_10_ (mm)	*ρ*_y_ (g·cm^−3^)
1	−1	−1	1	−1	1	0.265
2	−1	1	−1	1	1	0.256
3	1	−1	1	1	−1	0.279
4	1	1	−1	1	1	0.27
5	−1	1	1	1	−1	0.264
6	−1	1	1	−1	1	0.265
7	1	−1	−1	−1	1	0.276
8	1	1	−1	−1	−1	0.27
9	1	−1	1	1	1	0.287
10	−1	−1	−1	−1	−1	0.252
11	−1	−1	−1	1	−1	0.251
12	1	1	1	−1	−1	0.281

**Table 15:** Significance analysis of the Plackett–Burman test.

Source	Sum of Squares	Degree of Freedom	Mean Square	*F*	*p*	Contribution Rate (%)	Significance Ranking
Model	0.00141333	5	0.0002827	51.9184	<0.0001		
*x*_6_	0.0010083	1	0.0010083	185.204	<0.0001	69.35	1
*x*_7_	0.0000013	1	0.0000013	0.244898	0.638288	0.09	4
*x*_8_	0.000363	1	0.000363	66.6735	0.0001816	24.97	2
*x*_9_	0.0000003	1	0.0000003	0.0612245	0.812821	0.02	5
*x*_10_	0.0000403	1	0.0000403	7.40816	0.0345604	2.77	3
Residual	0.0000327	6	0.0000054				
Total	0.00144600	11					

**Table 16:** Design and results of the steepest ascent test.

Serial Number	*x*_6_ (N·m^−3^)	*x*_8_ (MPa)	*x*_10_ (mm)	*ρ*_y_ (g·cm^−3^)	Relative Error (%)
1	4.940 × 10^9^	2	0.240	0.252	5.97
2	5.093 × 10^9^	4	0.245	0.261	2.61
3	5.246 × 10^9^	6	0.250	0.267	0.37
4	5.398 × 10^9^	8	0.255	0.271	1.12
5	5.550 × 10^9^	10	0.260	0.278	3.73

**Table 17:** Coded levels of factors in the Box–Behnken response surface test.

Factor	Code		
	−1	0	1
Normal stiffness per unit area *x*_6_ (N·m^−3^)	5.093 × 10^9^	5.246 × 10^9^	5.398 × 10^9^
Critical normal stress *x*_8_ (MPa)	4	6	8
Contact radius *x*_10_ (mm)	0.245	0.250	0.255

**Table 18:** Design and results of the Box–Behnken response surface test.

Serial Number	Factor			*ρ*_y_ (g·cm^−3^)
	*x*_6_	*x*_8_	*x*_10_	
1	0	0	0	0.276
2	0	−1	−1	0.263
3	1	0	−1	0.266
4	−1	−1	0	0.239
5	−1	0	−1	0.242
6	−1	1	0	0.235
7	0	0	0	0.283
8	1	1	0	0.282
9	0	1	1	0.280
10	0	−1	1	0.270
11	0	1	−1	0.272
12	0	0	0	0.275
13	1	−1	0	0.259
14	0	0	0	0.285
15	1	0	1	0.288
16	0	0	0	0.277
17	−1	0	1	0.242

**Table 19:** Analysis of variance of the Box–Behnken response surface test.

Source	Sum of Squares	Degree of Freedom	Mean Square	*F*	*p*
Model	0.004728	9	0.0005253	40.72	<0.0001
*x*_6_	0.002377	1	0.002377	184.2	<0.0001
*x*_8_	0.0001892	1	0.0001892	14.66	0.006466
*x*_10_	0.0001514	1	0.0001514	11.73	0.01105
*x*_6_ *x*_8_	0.0001613	1	0.0001613	12.50	0.009524
*x*_6_ *x*_10_	0.0001177	1	0.0001177	9.125	0.01937
*x*_8_ *x*_10_	0.0000000625	1	0.0000000625	0.004844	0.9465
*x*_6_^2^	0.001440	1	0.001440	111.6	<0.0001
*x*_8_^2^	0.0002031	1	0.0002031	15.74	0.005409
*x*_10_^2^	0.000004821	1	0.000004821	0.3737	0.5603
Residual	0.00009031	7	0.00001290		
Lack-of-fit term	0.000007218	3	0.000002406	0.1158	0.9462
Pure error	0.00008309	4	0.00002077		
Total	0.004818	16			

**Table 20:** Discrete element parameters of leaf.

Object	Parameter	Value
Leaf	Density (g·cm^−3^)	0.908
	Poisson’s ratio	0.32
	Elastic modulus (MPa)	2.08
Leaf-Leaf	Static friction coefficient	0.56
	Dynamic friction coefficient	0.34
	Collision restitution coefficient	0.13
	Normal stiffness per unit area (N·m^−3^)	5.23 × 10^9^
	Tangential stiffness per unit area (N·m^−3^)	1.99 × 10^9^
	Critical normal stress (MPa)	4.189
	Critical tangential stress (MPa)	3.500
	Contact radius (mm)	0.251
Leaf-Steel plate	Static friction coefficient	0.52
	Dynamic friction coefficient	0.31
	Collision restitution coefficient	0.37

**Table 21:** Coded levels of factors in the Plackett–Burman test.

Factor	Code	
	−1	1
Normal stiffness per unit area *x*_11_ (N·m^−3^)	4.095 × 10^10^	4.153 × 10^10^
Tangential stiffness per unit area *x*_12_ (N·m^−3^)	1.540 × 10^10^	1.561 × 10^10^
Critical normal stress *x*_13_ (MPa)	8	18
Critical tangential stress *x*_14_ (MPa)	4	12
Contact radius *x*_15_ (mm)	0.24	0.26

**Table 22:** Design and results of the Plackett–Burman test.

Serial Number	*x*_11_ (N·m^−3^)	*x*_12_ (N·m^−3^)	*x*_13_ (MPa)	*x*_14_ (MPa)	*x*_15_ (mm)	*F*_2_ (N)
1	1	−1	1	1	1	3.14
2	1	1	−1	1	1	3.48
3	−1	−1	1	−1	1	2.98
4	−1	1	−1	1	1	3.40
5	−1	1	1	−1	1	3.39
6	1	−1	1	1	−1	2.87
7	−1	−1	−1	1	−1	2.56
8	1	−1	−1	−1	1	2.95
9	1	1	1	−1	−1	3.19
10	1	1	−1	−1	−1	3.16
11	−1	1	1	1	−1	3.10
12	−1	−1	−1	−1	−1	2.66

**Table 23:** Significance analysis of the Plackett–Burman test.

Source	Sum of Squares	Degree of Freedom	Mean Square	*F*	*p*	Contribution Rate (%)	Significance Ranking
Model	0.8786	5	0.1757	53.70	<0.0001		
*x*_11_	0.04083	1	0.04083	12.48	0.01233	4.53	3
*x*_12_	0.5461	1	0.5461	166.9	<0.0001	60.64	1
*x*_13_	0.01763	1	0.01763	5.389	0.05934	1.96	4
*x*_14_	0.004033	1	0.004033	1.233	0.3094	0.45	5
*x*_15_	0.2700	1	0.2700	82.51	<0.0001	29.98	2
Residual	0.01963	6	0.003272				
Total	0.8983	11					

**Table 24:** Design and results of the steepest ascent test.

Serial Number	*x*_11_ (N·m^−3^)	*x*_12_ (N·m^−3^)	*x*_15_ (mm)	*F*_2_ (N)	Relative Error (%)
1	4.0950 × 10^10^	1.54000 × 10^10^	0.240	2.81	8.47
2	4.1095 × 10^10^	1.54525 × 10^10^	0.245	2.94	4.23
3	4.1240 × 10^10^	1.55050 × 10^10^	0.250	3.05	0.65
4	4.1385 × 10^10^	1.55575 × 10^10^	0.255	3.11	1.30
5	4.1530 × 10^10^	1.56100 × 10^10^	0.260	3.19	3.91

**Table 25:** Coded levels of factors in the Box–Behnken response surface test.

Factor	Code		
	−1	0	1
Normal stiffness per unit area *x*_11_ (N·m^−3^)	4.1095 × 10^10^	4.1240 × 10^10^	4.1385 × 10^10^
Tangential stiffness per unit area *x*_12_ (N·m^−3^)	1.54525 × 10^10^	1.55050 × 10^10^	1.55575 × 10^10^
Contact radius *x*_15_ (mm)	0.245	0.250	0.255

**Table 26:** Design and results of the Box–Behnken response surface test.

Serial Number	Factor			*F*_2_ (N)
	*x*_11_	*x*_12_	*x*_15_	
1	1	0	−1	2.69
2	−1	−1	0	2.73
3	0	0	0	3.09
4	0	−1	0	2.85
5	0	−1	−1	2.46
6	0	1	1	3.65
7	0	0	0	3.03
8	1	1	0	3.49
9	−1	0	−1	2.53
10	0	0	0	3.03
11	−1	1	0	3.36
12	1	0	1	3.16
13	0	1	−1	2.92
14	1	−1	0	2.86
15	0	0	0	3.03
16	0	0	0	3.17
17	−1	0	1	3.16

**Table 27:** Analysis of variance of the Box–Behnken response surface test.

Source	Sum of Squares	Degree of Freedom	Mean Square	*F*	*p*
Model	1.583	9	0.1759	78.99	<0.0001
*x*_11_	0.02216	1	0.02216	9.948	0.01606
*x*_12_	0.7907	1	0.7907	355.0	<0.0001
*x*_15_	0.6094	1	0.6094	273.6	<0.0001
*x*_11_ *x*_12_	0.00000025	1	0.00000025	0.0001123	0.9918
*x*_11_ *x*_15_	0.006084	1	0.006084	2.732	0.1423
*x*_12_ *x*_15_	0.02958	1	0.02958	13.28	0.008235
*x*_11_^2^	0.001924	1	0.001924	0.8638	0.3836
*x*_12_^2^	0.01472	1	0.01472	6.609	0.03696
*x*_15_^2^	0.1114	1	0.1114	50.00	0.0001986
Residual	0.01559	7	0.002227		
Lack-of-fit term	0.001275	3	0.0004251	0.1188	0.9444
Pure error	0.01431	4	0.003579		
Total	1.599	16			

**Table 28:** Discrete element parameters of petiole.

Object	Parameter	Value
Petiole	Density (g·cm^−3^)	0.915
	Poisson’s ratio	0.33
Petiole-Petiole	Static friction coefficient	0.48
	Dynamic friction coefficient	0.29
	Collision restitution coefficient	0.29
Petiole-Steel plate	Static friction coefficient	0.50
	Dynamic friction coefficient	0.32
	Collision restitution coefficient	0.61
Large petiole	Elastic modulus (MPa)	16.45
	Normal stiffness per unit area (N·m^−3^)	4.112 × 10^10^
	Tangential stiffness per unit area (N·m^−3^)	1.552 × 10^10^
	Critical normal stress (MPa)	13
	Critical tangential stress (MPa)	8
	Contact radius (mm)	0.249
Small petiole	Elastic modulus (MPa)	14.43
	Normal stiffness per unit area (N·m^−3^)	3.608 × 10^10^
	Tangential stiffness per unit area (N·m^−3^)	1.356 × 10^10^
	Critical normal stress (MPa)	9.5
	Critical tangential stress (MPa)	6.5
	Contact radius (mm)	0.244

**Table 29:** Coded levels of factors in the Plackett–Burman test.

Factor	Code	
	−1	1
Normal stiffness per unit area *x*_16_ (N·m^−3^)	8.193 × 10^10^	8.403 × 10^10^
Tangential stiffness per unit area *x*_17_ (N·m^−3^)	2.968 × 10^10^	3.044 × 10^10^
Critical normal stress *x*_18_ (MPa)	15	25
Critical tangential stress *x*_19_ (MPa)	10	20
Contact radius *x*_20_ (mm)	0.24	0.26

**Table 30:** Design and results of the Plackett–Burman test.

Serial Number	*x*_16_ (N·m^−3^)	*x*_17_ (N·m^−3^)	*x*_18_ (MPa)	*x*_19_ (MPa)	*x*_20_ (mm)	*F*_3_ (N)
1	−1	−1	1	−1	1	4.92
2	−1	1	1	−1	1	4.83
3	1	1	1	−1	−1	4.25
4	1	1	−1	1	1	3.93
5	−1	−1	−1	−1	−1	3.05
6	1	−1	−1	−1	1	4.00
7	1	−1	1	1	−1	4.29
8	−1	−1	−1	1	−1	3.03
9	1	−1	1	1	1	5.25
10	1	1	−1	−1	−1	3.40
11	−1	1	−1	1	1	3.89
12	−1	1	1	1	−1	4.16

**Table 31:** Significance analysis of the Plackett–Burman test.

Source	Sum of Squares	Degree of Freedom	Mean Square	*F*	*p*	Contribution Rate (%)	Significance Ranking
Model	5.33697	5	1.06739	91.3603	<0.0001		
*x*_16_	0.128133	1	0.128133	10.9672	0.0161710	2.37	3
*x*_17_	0.0005333	1	0.0005333	0.0456491	0.837890	0.01	5
*x*_18_	3.41333	1	3.41333	292.154	<0.0001	63.17	1
*x*_19_	0.0008333	1	0.0008333	0.0713267	0.798357	0.02	4
*x*_20_	1.79413	1	1.79413	153.563	<0.0001	33.20	2
Residual	0.0701000	6	0.0116833				
Total	5.40707	11					

**Table 32:** Design and results of the steepest ascent test.

Serial Number	*x*_16_ (N·m^−3^)	*x*_18_ (MPa)	*x*_20_ (mm)	*F*_3_ (N)	Relative Error (%)
1	8.1930 × 10^10^	15	0.240	3.76	9.40
2	8.2455 × 10^10^	17.5	0.245	3.95	4.82
3	8.2980 × 10^10^	20	0.25	4.12	0.72
4	8.3505 × 10^10^	22.5	0.255	4.23	1.93
5	8.4030 × 10^10^	25	0.26	4.46	7.47

**Table 33:** Coded levels of factors in the Box–Behnken response surface test.

Factor	Code		
	−1	0	1
Normal stiffness per unit area *x*_16_ (N·m^−3^)	8.2455 × 10^10^	8.2980 × 10^10^	8.3505 × 10^10^
Critical normal stress *x*_18_ (MPa)	17.5	20	22.5
Contact radius *x*_20_ (mm)	0.245	0.250	0.255

**Table 34:** Design and results of the Box–Behnken response surface test.

Serial Number	Factor			*F*_3_ (N)
	*x*_16_	*x*_18_	*x*_20_	
1	1	0	1	4.46
2	0	0	0	4.20
3	1	−1	0	3.78
4	1	1	0	4.35
5	0	−1	−1	3.50
6	0	1	−1	4.04
7	−1	0	1	4.23
8	0	0	0	4.13
9	−1	1	0	4.21
10	0	0	0	4.10
11	1	0	−1	3.96
12	−1	0	−1	3.99
13	−1	−1	0	3.73
14	0	0	0	4.23
15	0	1	1	4.28
16	0	0	0	4.18
17	0	−1	1	3.77

**Table 35:** Analysis of variance of the Box–Behnken response surface test.

Source	Sum of Squares	Degree of Freedom	Mean Square	*F*	*p*
Model	0.995048	9	0.110561	43.7123	<0.0001
*x*_16_	0.0190125	1	0.0190125	7.51694	0.0288471
*x*_18_	0.551250	1	0.551250	217.947	<0.0001
*x*_20_	0.195313	1	0.195313	77.2204	<0.0001
*x*_16_ *x*_18_	0.00202500	1	0.00202500	0.800621	0.400626
*x*_16_ *x*_20_	0.0169000	1	0.0169000	6.68173	0.0362126
*x*_18_ *x*_20_	0.000225000	1	0.000225000	0.0889579	0.774165
*x*_16_^2^	0.0132042	1	0.0132042	5.22053	0.0562330
*x*_18_^2^	0.179546	1	0.179546	70.9870	<0.0001
*x*_20_^2^	0.0172463	1	0.0172463	6.81865	0.0348497
Residual	0.0177050	7	0.00252929		
Lack-of-fit term	0.00662500	3	0.00220833	0.797232	0.556239
Pure error	0.0110800	4	0.00277000		
Total	1.01275	16			

**Table 36:** Discrete element parameters of stem.

Object	Parameter	Value
Stem	Density (g·cm^−3^)	0.945
	Poisson’s ratio	0.38
	Elastic modulus (MPa)	33.19
Stem-Stem	Static friction coefficient	0.46
	Dynamic friction coefficient	0.23
	Collision restitution coefficient	0.32
Stem-Stem	Normal stiffness per unit area (N·m^−3^)	8.298 × 10^10^
	Tangential stiffness per unit area (N·m^−3^)	3.006 × 10^10^
	Critical normal stress (MPa)	22.42
	Critical tangential stress (MPa)	15
	Contact radius (mm)	0.248
Stem-Steel plate	Static friction coefficient	0.49
	Dynamic friction coefficient	0.28
	Collision restitution coefficient	0.69

## Data Availability

Data are contained within the article.
